# Impact of Social Vulnerability and Demographics on Ischemic Heart Disease Mortality in the United States

**DOI:** 10.1016/j.jacadv.2023.100577

**Published:** 2023-08-24

**Authors:** Ramzi Ibrahim, Mohammed Salih, Coco Victoria Gomez Tirambulo, Chelsea Takamatsu, Justin Z. Lee, David Fortuin, Kwan S. Lee

**Affiliations:** a Department of Internal Medicine, University of Arizona, Banner University Medical Center, Tucson, Arizona, USA; b Department of Cardiovascular Medicine, The Heart Hospital, Baylor University Medical Center, Plano, Texas, USA; c University of Arizona College of Medicine-Tucson, Tucson, Arizona, USA; d Department of Cardiovascular Medicine, Cleveland Clinic, Cleveland, Ohio, USA; e Department of Cardiovascular Medicine, Mayo Clinic, Phoenix, Arizona, USA

**Keywords:** disparities, epidemiology, ischemia, population, social

## Abstract

**Background:**

Cardiovascular disease is a leading cause of morbidity and mortality, largely dominated by ischemic heart diseases (IHDs). Social determinants of health, including geographic, psychosocial, and socioeconomic factors, influence the development of IHD.

**Objectives:**

This study aimed to evaluate yearly trends and disparities in IHD mortality and to assess the impact of social vulnerability.

**Methods:**

We performed cross-sectional analyses using United States county-level mortality data and social vulnerability index (SVI) obtained from the Centers for Disease Control and Prevention databases. Age-adjusted mortality rates (AAMRs) per 100,000 population were compared between aggregated U.S. county groups, stratified by demographic information and SVI quartiles. Log-linear regression models were used to identify mortality trends from 1999 to 2020, with inflection points determined through the Monte-Carlo permutation test.

**Results:**

We identified a total of 9,108,644 deaths related to IHD between 1999 and 2020. Overall AAMR decreased from 194.6 in 1999 to 91.8 in 2020. Males (AAMR: 161.51) and Black (AAMR: 141.49) populations exhibited higher AAMR compared to females (AAMR: 93.16) and White (AAMR: 123.34) populations, respectively. Disproportionate AAMRs were observed among nonmetropolitan (AAMR: 136.17) and Northeastern (AAMR: 132.96) regions. Counties with a higher SVI experienced a greater AAMR, with a cumulative excess of 20.91 deaths per 100,000 person-years associated with increased social vulnerability.

**Conclusions:**

Despite a decline in IHD mortality from 1999 to 2020, disparities persisted among racial, gender, and geographic subgroups. A higher SVI was linked to increased IHD mortality. Policy interventions should prioritize integrating the SVI into health care delivery systems to effectively address these disparities.

Cardiovascular disease (CVD) is a leading cause of morbidity and mortality in the United States, dominated by 126 million people globally living with ischemic heart disease (IHD).^1^ Despite the progressive decline in mortality related to IHD in the past few decades, it remains a major burden to health care.^1^ Risk factor prevalence continues to increase, largely contributed by social determinants of health.^1^ These determinants originate from a variety of social constructs including geographic, socioeconomic, and dietary factors. Identifying gaps in care among these social determinants remains a vital objective of population-level research.

The Centers for Disease Control and Prevention (CDC)/Agency for Toxic Substances and Disease Registry (ATSDR) has integrated key social aspects that quantify the potential of adversity on U.S. communities by external stressors, termed the social vulnerability index (SVI).^2^ This scoring system has been utilized by public health officials to identify communities that may need support before, during, and after an external stressor such as human-caused disasters, disease outbreaks, and natural disasters. Moreover, this framework allows researchers to identify communities with greater susceptibility to adverse outcomes from a multitude of illnesses. In the present study, we characterized mortality trends, explored disparities among gender, racial, and geographic subgroups, and analyzed the impact of social vulnerability on IHD mortality in the United States.

## Methods

We obtained mortality data from the CDC Wide-Ranging Online Data for Epidemiologic Research (WONDER) database which utilizes the National Vital Statistics System to capture all death-related information from death certificates in the United States.^3^ Death certificate information included the underlying cause of death, the multiple causes of death, and demographic data. Underlying cause of death, defined by the World Health Organization, was described as the main diagnosis that led to death or initiated the sequence of events that led to death. The multiple causes of death, defined by the CDC, were described as the contributors to death. When more than one diagnosis is included in the death certificate, the underlying cause was determined by the sequence of diagnoses on the certificate. All included diagnoses in this article are based on the International Classification of Diseases-10th Revision (ICD-10). Demographic information including gender (ie, male vs female), race (ie, White, Black, American Indian/Alaska Native, and Asian/Pacific Islander), and geographic data were made available. Race information was reported by the funeral director if available, usually provided by a surviving next of kin or based on observation if no informant is available. Geographic information included the U.S. census regions (ie, Northeast, Midwest, South, and West). We also used the National Center for Health Statistics 2013 Urban–Rural Classification Scheme, aggregating county-level data into either metropolitan or nonmetropolitan groups.

We queried the CDC ATSDR database for the SVI 2018 data release, which is derived from 5-year estimates of the American Community Survey (2014-2018).^2^ The data sets are named based on the latest year of data within the 5-year American Community Survey aggregated data. To ensure accuracy at the census tract/county level, smaller population sizes necessitate the use of 5-year estimates instead of 1-year estimates. This database outlines every U.S. census tract and county based on 15 social attributes and groups them into 4 themes (Supplemental Table 1). The themes included are household composition and disability (≥65 years of age; ≤17 years of age; ≥5 years of age with a disability; single-parent households), housing type and transportation (multiunit structure, mobile home, crowding, no vehicle, group quarters), minority status and language (minority; speak English “less than well”), and socioeconomic status (below poverty, unemployed, income, no high school diploma). Percentile rankings were calculated for overall SVI within each U.S. county, ranging from 0 to 1, with higher values exhibiting greater social vulnerability than lower values.

Institutional Review Board approval was not required as the data collected are publicly available in the data repositories in a deidentified format.

### Statistical analysis

All mortality data related to IHD (ICD-10 codes: I20-I25) as the underlying cause of death were queried from 1999 until 2020. Data were obtained for the overall population and for gender, race, and geographic subgroups. Quantified measures included absolute death rate count, crude mortality rates, age-adjusted mortality rates (AAMRs), and respective 95% CIs. AAMR was calculated per 100,000 population adjusted to the U.S. population in the year 2000. We conducted an analysis of AAMR trends over time using log-linear regression models through Joinpoint Regression (National Cancer Institute).^4^, ^5^, ^6^, ^7^, ^8^, ^9^, ^10^ To identify segments where the trend significantly changes, we located joinpoints and calculated annual percentage changes with 95% CIs at these points using the Monte Carlo permutation test.^4^^,^^6^, ^7^, ^8^ To determine the average annual percentage change (AAPC), we calculated weighted averages of the annual percentage change. We followed the National Cancer Institute's recommendation of allowing up to 4 joinpoints given the 22 yearly AAMR estimates in our analysis.^9^ To determine if the slope of the change was significantly different than zero, we conducted 2-tailed *t*-testing. We considered the AAPC significant if the slope was significantly different than zero, with statistical significance set at *P* < 0.05.

Four quartiles were used for percentile rankings of overall SVI among all U.S. counties (0-0.25 as first quartile and least vulnerable, >0.25-0.50 as second quartile, >0.50-0.75 as third quartile, and >0.75-1.00 as fourth quartile and most vulnerable). Within these quartiles, we estimated AAMR for cumulative and subgroups (ie, gender, race, and geographic) from the years 2014 to 2018. Risk ratio and associated 95% CIs were estimated by comparing the AAMR between the first and fourth quartiles by Poisson univariable regression; 95% CIs that did not cross 1.0 were considered statistically significant.

Data analysis and visualization were completed using Stata (Stata Statistical Software: Release 17.0; StataCorp LLC).

## Results

**Figure 1:**
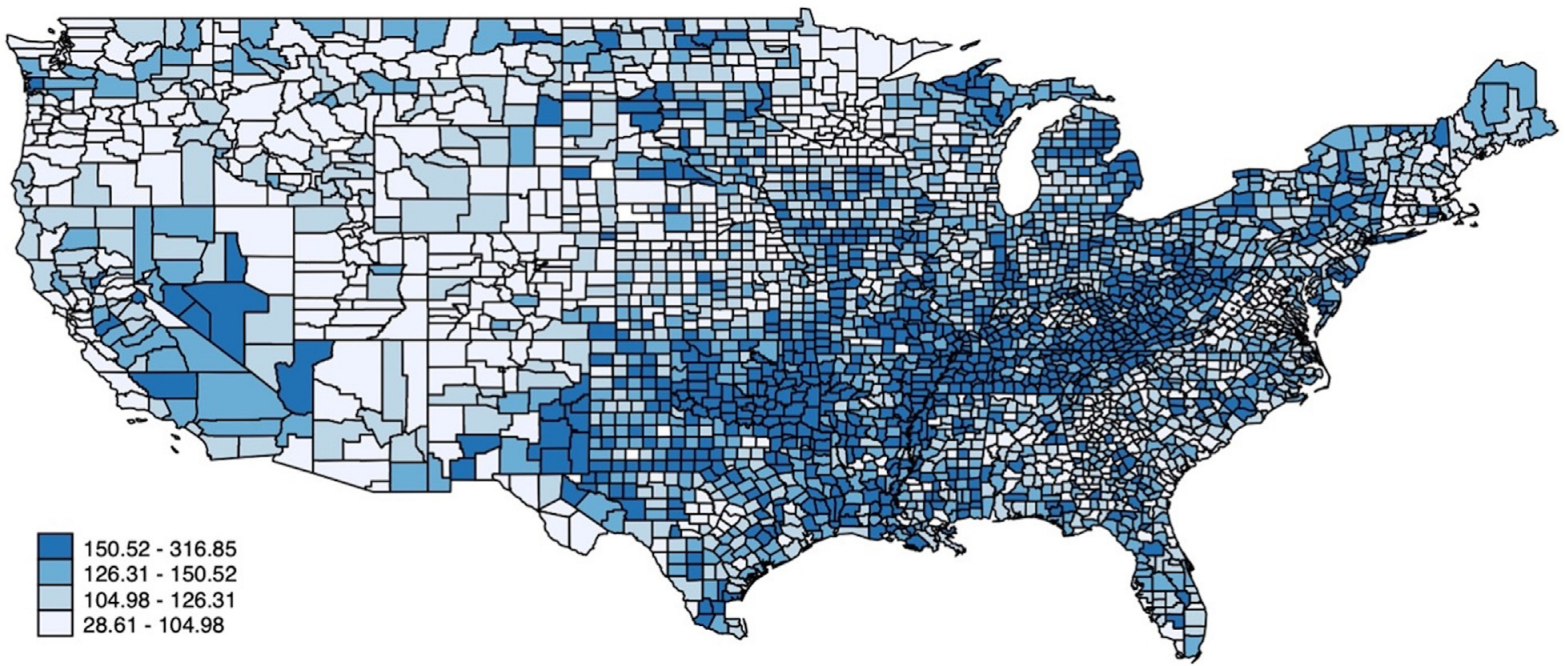
**Choropleth Map of AAMRs Across the U.S. Counties** Map depicts overall U.S. county-level AAMR per 100,000 population. AAMR = age-adjusted mortality rates.

**Figure 2:**
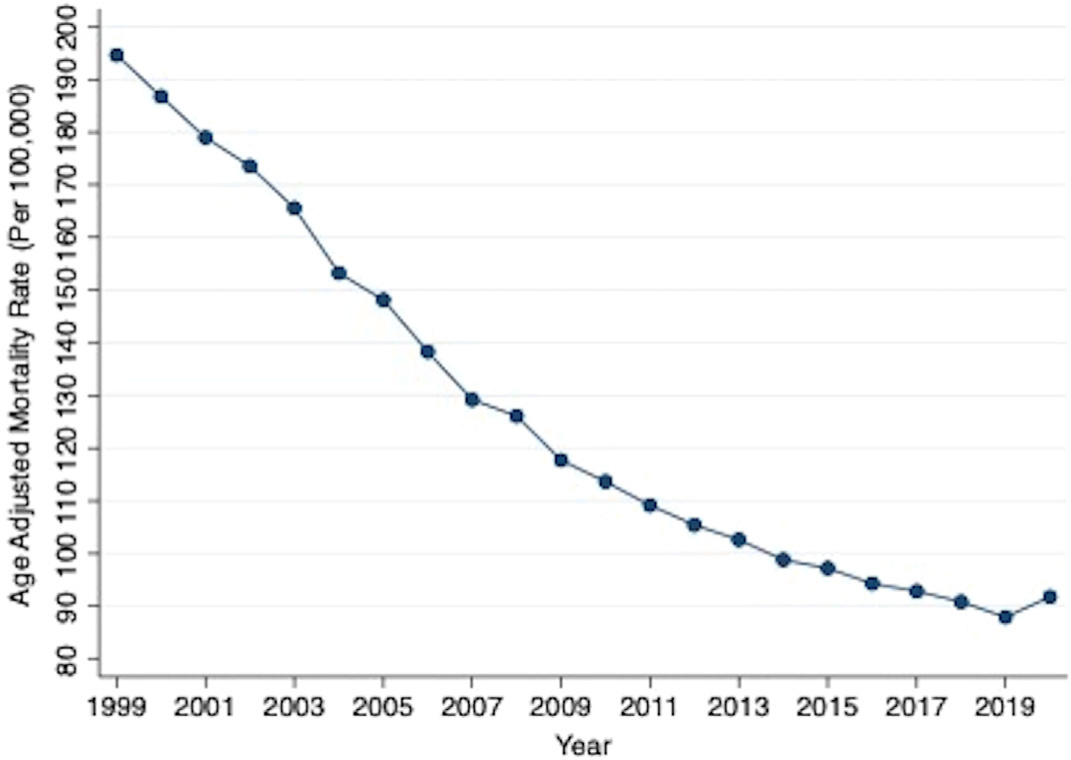
**AAMR per 100,000 Population Related to Ischemic Heart Diseases** Yearly connected plot of overall AAMR between 1999 and 2020. Overall AAPC: −3.6% (95% CI: −3.9 to −3.2), *P* < 0.001. AAMR = age-adjusted mortality rates; AAPC = average annual percentage change.

There were a total of 9,108,644 deaths related to IHD from 1999 to 2020 in the United States (Figure 1). All mortality rates (crude and age-adjusted), CIs, and AAPC are shown in Supplemental Table 2. There was a decrease in overall crude and AAMR from 1999 to 2020. AAMR decreased from 194.6 [95% CI: 194.07-195.12] in 1999 to 91.8 [95% CI: 91.5-92.09] in 2020 (Figure 2). The AAPC was −3.6% [95% CI: −3.9 to −3.2] (Central Illustration).

**Central Illustration:**
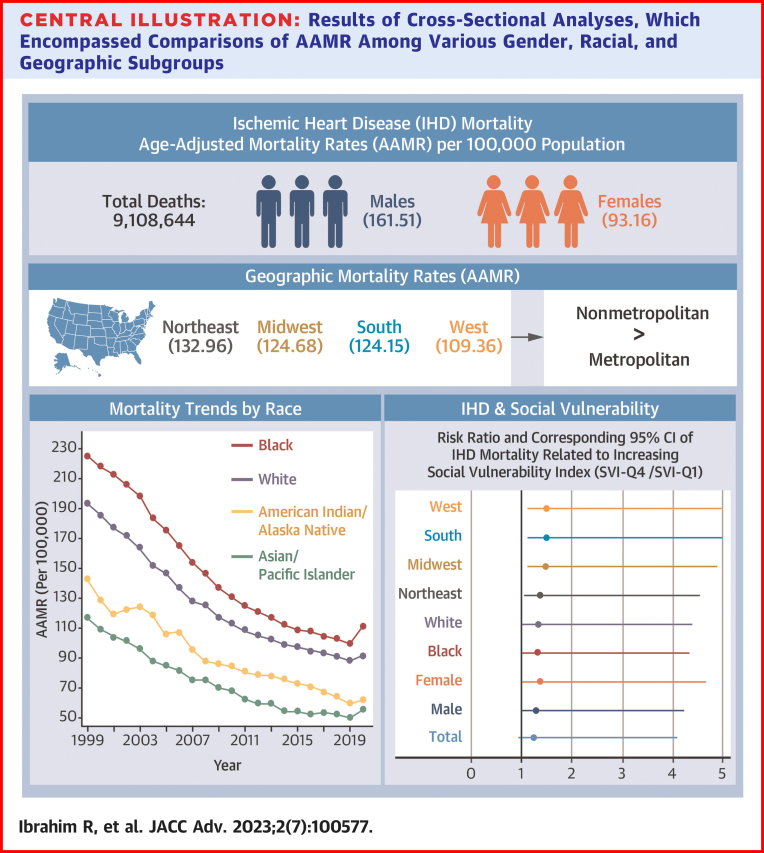
**Results of Cross-Sectional Analyses, Which Encompassed Comparisons of AAMR Among Various Gender, Racial, and Geographic Subgroups** To visually depict the risk ratios, we utilized a forest plot that highlights the variations between the least and most socially vulnerable quartiles of U.S. counties. AAMR = age-adjusted mortality rates.

All identified inflection points are illustrated in Supplemental Table 3. Our study revealed that a significant inflection point occurred between 2009 and 2012 for most of the populations included in the study. However, the Asian/Pacific Islander and American Indian/Alaska Native subpopulations were exceptions to this trend and did not experience a statistically significant inflection point in these same years.

**Figure 3:**
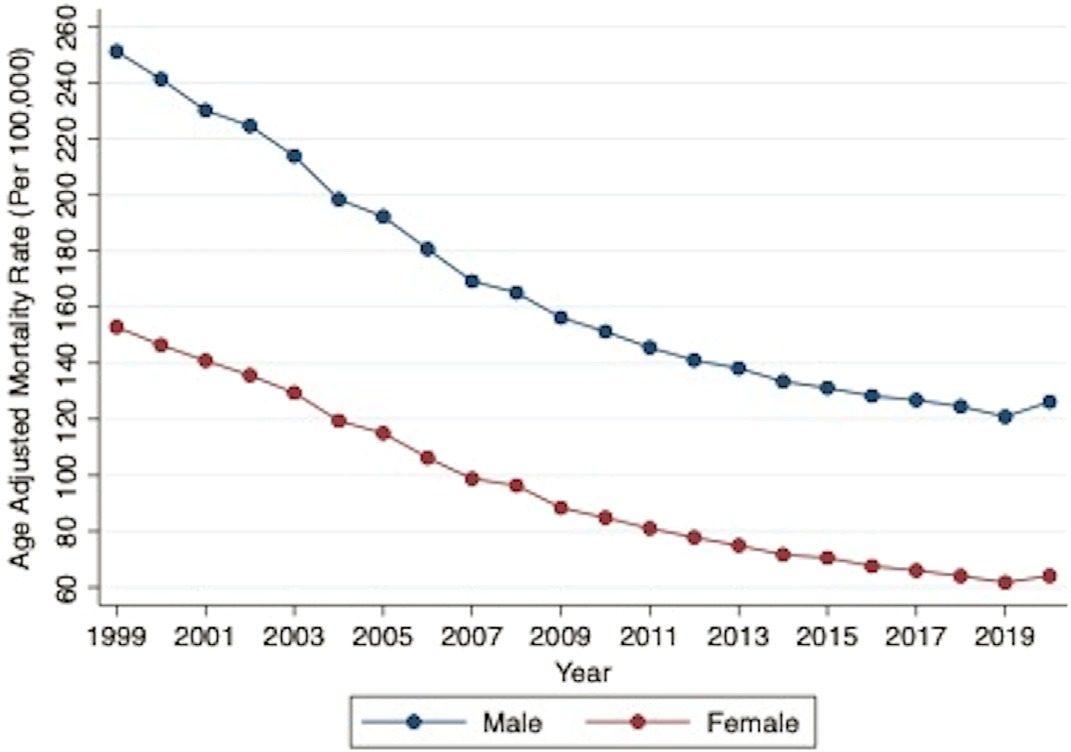
**AAMR per 100,000 Population Related to Ischemic Heart Diseases by Gender** Yearly connected plot of overall AAMR by gender subgroups between 1999 and 2020. Male AAPC: −3.5% (95% CI: −3.7 to −3.2), *P* < 0.001, Female AAPC: −4.1% (95% CI: −4.5 to −3.7), *P* < 0.001. AAMR = age-adjusted mortality rates; AAPC = average annual percentage change.

The crude mortality and AAMR decreased from 1999 to 2020 for both male and female populations (Figure 3). The overall AAMR for male populations (161.51 [95% CI: 161.37-161.66]) was higher compared to female populations (93.16 [95% CI: 93.07-93.25]). The decreasing AAPC was similar among both male (−3.5% [95% CI: −3.7 to −3.2]) and female (−4.1% [95% CI: −4.5 to −3.7]) populations.

**Figure 4:**
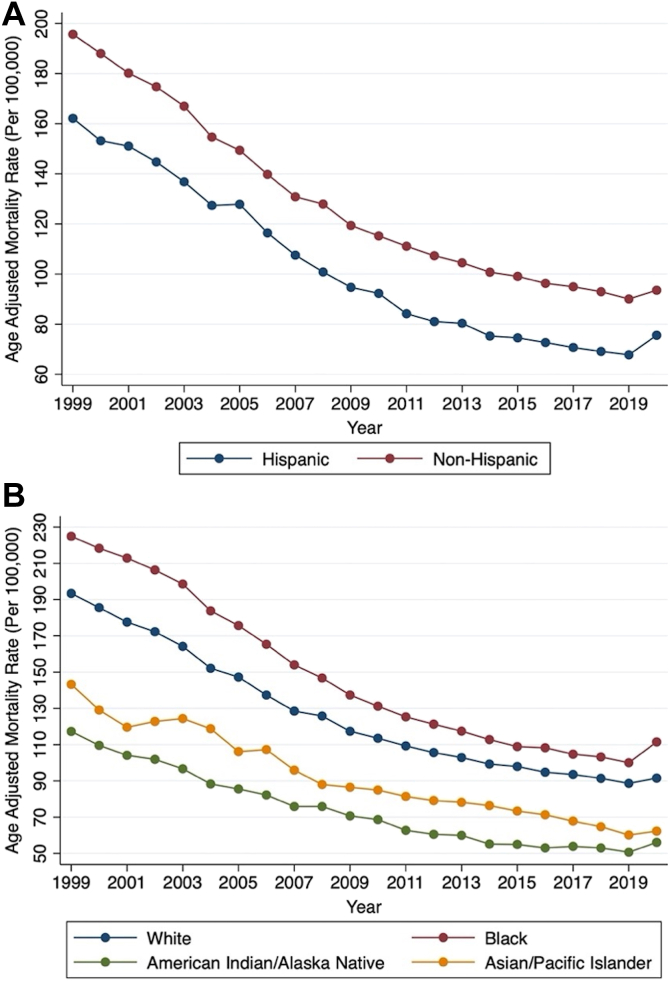
**AAMR per 100,000 Population Related to Ischemic Heart Diseases by Racial Subgroups** **(A)** Yearly connected plot of overall AAMR by Hispanic and non-Hispanic subgroups between 1999 and 2020. **(B)** Yearly connected plot of overall AAMR by race between 1999 and 2020. Hispanic AAPC: −3.7% (95% CI: −4.2 to −3.1), *P* < 0.001. Non-Hispanic AAPC: −3.5% (95% CI: −3.9 to 3.1), *P* < 0.001. White AAPC: −3.6% (95% CI: −4.0 to −3.2), *P* < 0.001. Black AAPC: −3.4% (95% CI: −3.7 to −3.1), *P* < 0.001. Asian/Pacific Islander AAPC: −3.6% (95% CI: −4.0 to −3.2), *P* < 0.001. American Indian/Alaska Native AAPC: −3.7% (95% CI: −4.8 to −2.6), *P* < 0.001. AAMR = age-adjusted mortality rates; AAPC = average annual percentage change.

AAMR was greater within non-Hispanic populations (125.2; 95% CI: 125.12-125.29) compared to Hispanic populations (92.94 [95% CI: 92.67-93.21]). Both groups had a similar yearly AAPC (−3.5% [95% CI: −3.9 to −3.1] and −3.7% [95% CI: −4.2 to −3.1], respectively) (Figure 4A). All 4 races included in this analysis had a downtrending crude and AAMR (Figure 4B). Overall, AAMR was most pronounced among Black populations (141.49 [95% CI: 141.2-141.78]), followed by White populations (123.34 [95% CI: 123.25-123.43]), American Indian/Alaska Native populations (85.1 [95% CI: 84.24-85.96]), and Asian/Pacific Islander populations (66.88 [95% CI: 66.57-67.20]). However, the AAPC was similar among all 4 racial subgroups.

**Figure 5:**
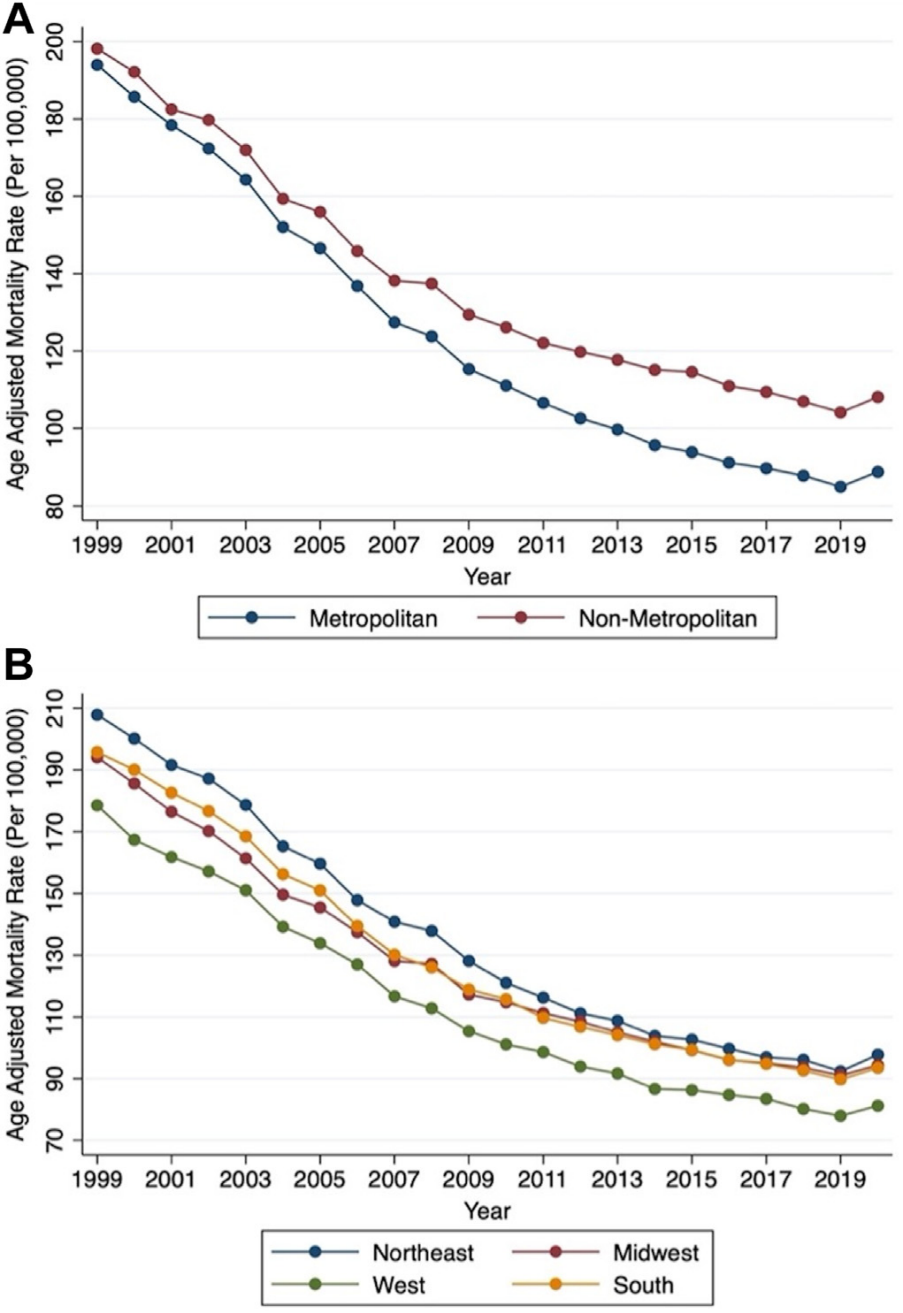
**AAMR per 100,000 Population Related to Ischemic Heart Diseases by Geographic Subgroups** **(A)** Yearly connected plot of overall AAMR by metropolitan and nonmetropolitan subgroups between 1999 and 2020. **(B)** Yearly connected plot of overall AAMR by U.S. census regions between 1999 and 2020. Metropolitan AAPC: −3.7% (95% CI: −4.1 to −3.3), *P* < 0.001. Nonmetropolitan AAPC: −3.1% (95% CI: −3.3 to −2.8), *P* < 0.001. Northeast AAPC: −3.6% (95% CI: −4.0 to −3.1), *P* < 0.001. Midwest AAPC: −3.5% (95% CI: −3.8 to −3.1), *P* < 0.001. South AAPC: −3.5% (95% CI: −3.9 to −3.1), *P* < 0.001. West AAPC: −3.9% (95% CI: −4.2 to −3.6), *P* < 0.001. AAMR = age-adjusted mortality rates; AAPC = average annual percentage change.

Geographic variability in IHD mortality did exist among the U.S. census regions and between nonmetropolitan and metropolitan regions. There was a downtrend in crude and AAMR for all geographic subgroups from 1999 to 2020 (Figures 5A and 5B). For example, the overall AAMR for nonmetropolitan regions (136.17 [95% CI: 135.97-136.37]) was higher compared to metropolitan regions (120.42 [95% CI: 120.33-120.51]); however, a similar AAPC was observed among both regions (−3.1% [95% CI: −3.3 to −2.8] and −3.7% [95% CI: −4.1 to −3.3], respectively). The Northeast (132.96 [95% CI: 132.77-133.15]) held the greatest burden of AAMR, followed by the Midwest (124.68 [95% CI: 124.51-124.85]), South (124.15 [95% CI: 124.01-124.28]), and the West (109.36 [95% CI: 109.19-109.52]). All 4 U.S. census regions had a similarly decreasing AAPC.

**Figure 6:**
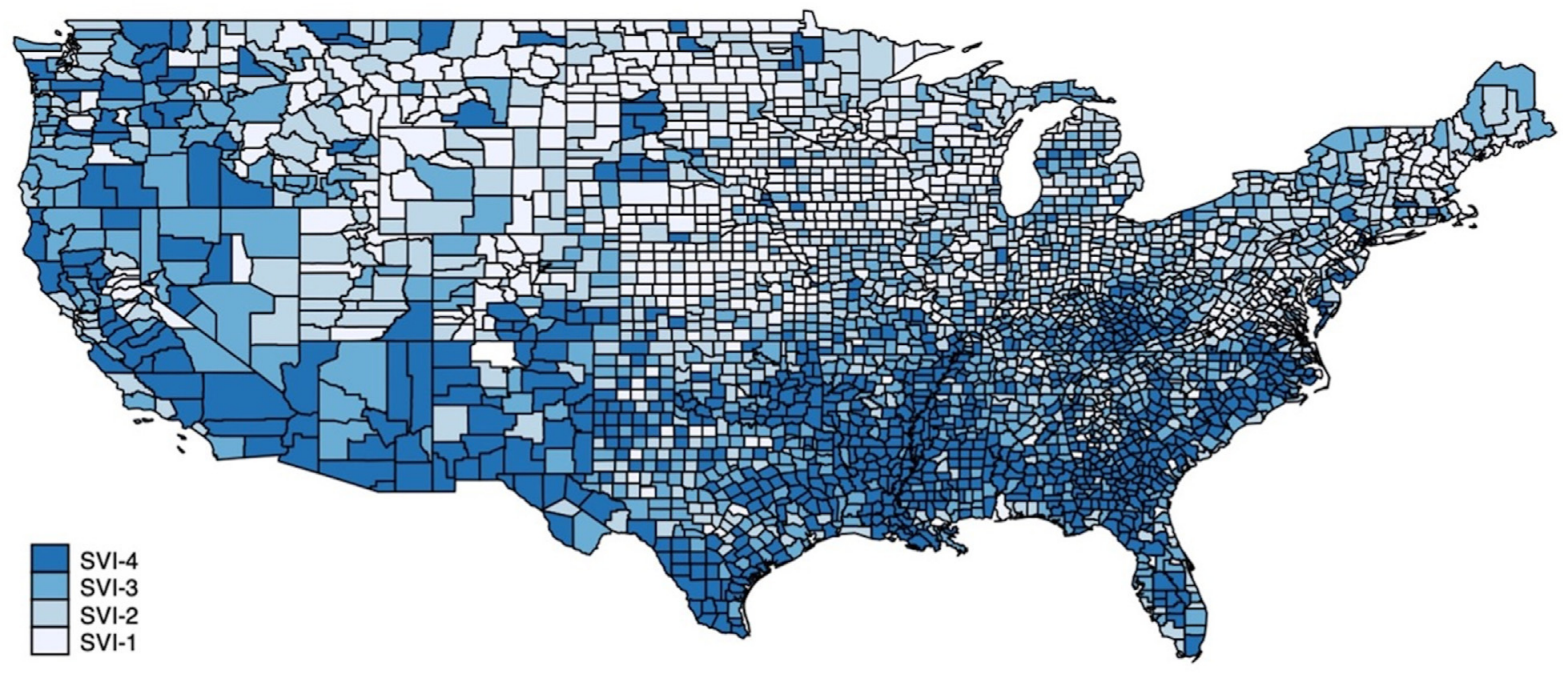
**Choropleth Map of Social Vulnerability Index Quartiles Across the U.S. Counties** Map depicts overall U.S. county-level social vulnerability index quartiles.

All U.S. counties, regardless of population size and total death count, were aggregated into 4 quartiles related to SVI (SVI-Q1 to SVI-Q4) (Figure 6). Social attributes reported in the SVI ranking by the CDC/ATSDR between 2014 and 2018 were reported as median (IQR), displayed in Supplemental Table 4. AAMR for the years 2014 to 2018 were calculated for all 4 SVI quartiles using cumulative mortality rates and within subgroups, displayed in Supplemental Table 5. Compared to SVI-Q1, the AAMR in SVI-Q4 was statistically higher in all analyses. For example, overall AAMR was lower in SVI-Q1 (90.39 [95% CI: 89.62-91.16]) compared to SVI-Q4 (111.30 [95% CI: 110.98-111.62]), with higher social vulnerability accounting for 20.91 excess deaths per 100,000 person-years. The risk ratio was >1 for all groups (cumulative and within subgroups); however, only a minority of the CIs included 1 [6 out of the 15]. There was an average of 1.4 times greater mortality risk related to IHD among the most socially vulnerable compared to the least socially vulnerable.

## Discussion

Our analysis of 9,108,644 deaths related to IHD from 1999 to 2020 revealed: 1) there is an overall decreasing death count related to IHD; 2) presence of racial and gender disparities, specifically noting a higher AAMR among Black individuals and males; 3) regional disparities in AAMR within nonmetropolitan areas and Northeastern regions; and 4) the SVI is associated with increasing mortality rates related to IHD. These results hold significant importance for public health policy, as they contribute meaningful epidemiological insights into the overall mortality of IHD.

Our finding of declining mortality rates related to IHD is consistent with other studies.^11^ This can be explained by improvement in health care delivery systems and increasing availability of preventative and treatment modalities. For example, the availability of therapeutic technologies including cardiac catheterization laboratories remains vital to these findings, likely contributing to the decrease in mortality. Additionally, advancements in pharmacological therapy for prevention and management of IHD have also showed mortality benefit in the survival of these patients.^12^

Our study uncovered significant inflection points occurring around 2009 to 2012, impacting most populations examined, both overall and among subpopulations. Notably, this pattern was not observed in the Asian/Pacific Islander and American Indian/Alaska Native subpopulations. These findings are consistent with multiple analyses that have reported similar deflection points in 2010 or 2011, resulting in either a slowed downtrend or a slight increase in cardiovascular mortality, depending on the population studied.^13^, ^14^, ^15^ Several factors may have contributed to the plateau and slight increase in mortality, including the expanded use of electronic health records to record certain CVDs, the implementation of readmission reduction programs, and the rising prevalence of CVD risk factors.^15^, ^16^, ^17^, ^18^ Moreover, these trends align with a period that witnessed increased hospitalization rates for heart failure among younger adults, which corresponds to our study findings.^19^ Despite the progress made in targeted health policy interventions and improved clinical risk management during the early 2000s, our study underscores the need for continued efforts to effectively implement these strategies and achieve additional survival gains.

A disproportionate mortality rate among non-Hispanic and Black populations was observed. Multiple studies have identified racial and ethnic minorities to have a higher predisposition to IHD.^20^, ^21^, ^22^, ^23^, ^24^ For example, Black populations are at greater risks of myocardial infarction, heart failure, and hospitalizations related to acute coronary syndrome, and are more likely to have symptoms and functional impairment from coronary artery disease.^21^ These populations are also known to have a higher prevalence of associated risk factors, including hypertension and diabetes mellitus, and are less likely to receive care that meets current standard guidelines.^21^

We identified significant geographic heterogeneity in the burden of IHD mortality. For example, nonmetropolitan areas had higher mortality rates compared to metropolitan areas. The Northeast had the highest AAMR among the U.S. census regions, followed by the Midwest, South, and West regions. This variation is likely attributable to demographic and economic/social conditions.^25^ Social determinants of health and lower economic status are associated with higher risks of atherosclerotic CVD and myocardial infarction, and an overall lower rate of observed cardiovascular health.^21^^,^^26^ Disadvantaged neighborhoods have worse cardiometabolic health and higher risk of IHD.^27^^,^^28^ This is coupled with higher risk factor prevalence including tobacco use, alcohol use, obesity, and physical inactivity. Healthier dietary options and accessibility to fresh fruits and vegetables remain limited in certain regions, constituting another barrier to a healthier lifestyle.^29^ Optimal health status is positively correlated with access to quality care, affordable medications, adherence to guideline-directed medical therapy, and a supportive cultural environment.^28^ Access to preventative care including primary care physicians and cardiologists remains a problem in many regions in the United States, particularly in rural and nonmetropolitan areas.^21^

In our analyses, higher rates of age-adjusted mortality were observed within U.S. counties affected by greater social vulnerability, overall and across all subgroups. Khan et al^30^ evaluated premature cardiovascular mortality in relation to the SVI among individuals under the age of 65 years. They found that a higher SVI was associated with increased mortality rates related to premature CVD, including IHD. Similarly, our study revealed that SVI-impacted individuals of all ages, with the majority being over 65 years, leading to higher IHD-related mortality. However, our study goes beyond Khan et al in several other ways. We investigated the impact of SVI on IHD mortality in various ethnic backgrounds, including American Indian/Alaska Native and Asian/Pacific Islander populations, which were not examined in the previous study. Additionally, we also stratified results by the U.S. census regions (ie, Northeast, Midwest, South, and West), which were not included in Khan et al. Furthermore, our study also explored a 22-year mortality trend related to IHD.

Multiple studies have contributed to the evaluation of mortality trends related to IHD.^14^^,^^31^, ^32^, ^33^ For instance, Essa et al^31^ found a greater relative decrease in annual AAMR in women compared to men, whereas our study revealed a similar decreasing AAMR in both males and females. We also expand upon the findings of Essa et al by incorporating an assessment of IHD mortality trends based on geography, specifically U.S. census regions and urbanization, rather than focusing solely on U.S. states. Similarly, Shah et al^14^ investigated overall cardiovascular death, including IHD-specific ICD10 codes, while our study specifically focused on IHD mortality trends and disparities. Moreover, our analysis expanded on the work of Shah et al^14^ by examining IHD mortality trends among various racial backgrounds, such as Asian/Pacific Islanders and American Indian/Alaska Native populations. In another study, Khan et al^33^ examined rural and urban differences in cardiovascular mortality, including subgroups with IHD ICD10 codes. In contrast, our study focused on subpopulations based on gender, racial/ethnic backgrounds, and U.S. census regions, rather than solely focusing on urbanization. Lastly, Sidney et al^32^ analyzed U.S. mortality trends until 2015, with a focus on CVD subgroups that included IHD mortality ICD10 codes. However, our study extended the analysis until 2020 and included an evaluation of regional disparities based on U.S. census regions and urbanization, aspects that were not addressed in Sidney et al.^32^ By incorporating these unique elements, our study emphasizes the impact of race/ethnic backgrounds, urbanization, and regional disparities. This sets our findings apart from other recent studies and contributes to a more nuanced understanding of IHD mortality.

Multiple scoring systems have been introduced to quantify the impact of social determinants of health. For example, the area deprivation index focuses on socioeconomic deprivation but lacks in the many variables included within the SVI.^34^ The SVI encompasses a comprehensive global assessment of social vulnerability, unlike the other scoring systems, including English insufficiency, elderly and younger populations, and disabilities. The SVI remains a valuable tool for researchers as it includes multiple determinants of cardiovascular health and is associated with an increasing prevalence of chronic comorbidities including diabetes mellitus, hypertension, hyperlipidemia, smoking, atherosclerotic CVD, and chronic kidney disease, ultimately leading to increased CVD and mortality rates.^35^ Consistently, our study revealed disproportionate IHD mortality among populations affected by a greater SVI.

Population-level risk assessment of CVD remains a vital stepping stone to identifying inequality in health care. Social determinants of health and systemic and structural racism are major contributors to existing disparities.^36^ Not only do these factors contribute to disproportionate health care utilization and mortality among certain populations but also to significant economic burden.^21^ Our findings carry significant implications. Traditional risk factor and prognostication algorithms need to be continuously updated to include the many social determinants of health.^37^^,^^38^ Policy efforts are warranted to integrate factors related to social vulnerability into existing health care delivery systems, enabling providers and hospital systems to target these predisposed populations.^39^, ^40^, ^41^ This includes expansion of current insurance programs such as Medicaid to mitigate gaps in care and narrow existing disparities.^41^ Many of these populations are disadvantaged and special workforce training should be provided to providers treating these populations to assist with informed care.

### Study limitations

Limitations to our study include the use of death certificates to capture all mortalities in the United States, which are subjected to inaccuracies. Covariates related to the management of included individuals are not available in the specified databases. Additionally, reverse association remains a possibility. For example, increasing IHD mortality may also lead to worsening social vulnerability. Therefore, causality could not be established in our study given the cross-sectional design. Utilizing the SVI does not take into account food insecurity, barriers to health care access, and community contextual factors. Lastly, confounding variables, other than age, were not taken into account.

## Conclusions

Although IHD mortality has decreased from 1999 to 2020, disparities related to IHD mortality exist among gender, racial, and geographic subgroups. The SVI is associated with higher rates of IHD mortality. Risk algorithms used by providers and health care delivery systems need to be continually adjusted to account for social determinants of health. Addressing disparities is most effective when all aspects of socioeconomic factors are taken into consideration.

## Funding support and author disclosures

The authors have reported that they have no relationships relevant to the contents of this paper to disclose.

PERSPECTIVES **COMPETENCY IN PATIENT CARE:** Through our cross-sectional analyses, we discovered that Black and male populations faced the highest risk of mortality associated with ischemic heart diseases. Moreover, our findings revealed that residents residing in U.S. counties characterized by higher social vulnerability experienced worse mortality outcomes related to ischemic heart disease. These insights highlight the need for targeted interventions and tailored patient care strategies to address the specific risks and challenges faced by these vulnerable populations.

**TRANSLATIONAL OUTLOOK:** It is crucial to incorporate demographic details and the social vulnerability index within health care delivery systems and risk algorithms. This integration will enable the identification of populations at a higher risk of adverse outcomes related to ischemic heart disease.
