# Supplementary material for: Impact of Social Vulnerability and Demographics on Ischemic Heart Disease Mortality in the United States
Source: JACC Adv. 2023 Aug 24;2(7):100577. doi: 10.1016/j.jacadv.2023.100577 (PMC11198229; doi:10.1016/j.jacadv.2023.100577)
Supplement: Supplementary data [file mmc1.docx]

**SUPPLEMENTAL APPENDIX**

**Supplemental Methods**

All mortality data used in our analysis was queried from the National Vital Statistics System which captures all mortality data in the United States (US) for US residents based on death certificate information. Merged with the CDC WONDER database, we were able to obtain this data through their publicly available and de-identified repository online. This database includes all underlying causes of death, multiple causes of death, and related demographic information including gender, geographic information, and race. We specifically focused on ischemic heart diseases (IHD) as the underlying cause of death. Per the CDC WONDER database, which uses the definition recommended by the WHO, underlying cause of death is defined as the disease process that directly led to mortality or initiated the events that ultimately led to mortality. Underlying cause of death was determined by the condition entered by the provider on the cause of death section of the death certificate, or if more than one cause were entered, the underlying cause of death was determined by the sequence of conditions on the certification. Specific ICD-10 codes for ischemic heart diseases (IHD) that were used include: I20 – I25. Crude mortality rates were obtained for IHD as underlying cause of death with additional adjustments for age. Age-adjusted mortality rates (AAMR) values were obtained for cumulative population rates from 1999 until 2020 and for subgroups including gender, race, and geographic information. Gender included male and female. Race included White populations, Black populations, American Indian/Alaska Native populations, and Asian/Pacific Islander populations. Race and Hispanic origin are reported by the funeral director if provided by an informant which is often the deceased’s next of kin. If no informant was available, racial origin was made by basis of observation. Asian and Pacific Islander category included Hawaiian, Japanese, Filipino, Chinese, and Other Asian or Pacific Islanders. American Indian or Alaska Native category included Central, North, and South American Indians, Aleuts, and Eskimos. Race and Hispanic origin were reported on death certificates, standards that have been set forth by the Office of Management and Budget. If Hispanic origin was not reported on a death certificate, it was specifically coded as “not stated” on the CDC WONDER database and excluded in this analysis. Geographic information included US Census regions which is divided into Northeast, Midwest, West, and South regions. Additionally, using the 2013 NCHS Urban-Rural Scheme for Counties Urbanization Criteria, we then evaluated AAMR among metropolitan (Large central metro, large fringe metro, medium metro, small metro) and non-metropolitan regions (Micropolitan and NonCore regions).

Population estimates included by the CDC WONDER Database are based on the US Census Bureau estimates of US national, state, and county resident populations. All AAMR were adjusted to the year 2000 US population. AAMR in this database are weighted averages of age-specific death rates, where the weight represents a fixed population by age. Age-adjustments were done to remove effects of age from the crude rates of mortality.

We obtained data regarding the social vulnerability index (SVI) in all US counties from the CDC Agency for Toxic Substances and Disease Registry. This scoring system assesses the vulnerability of all US census tracts and counties using data from the American Community Survey. Within this scoring system, there are 15 social factors that are observed and grouped into four themes (**Table S1**). Five-year estimates of data used for this analysis were specifically from the American Community Survey, 2014 – 2018 (**Table S4**). US census track was defined as a subdivision of counties for which the census collects data. Overall, each county receives a ranking for overall social vulnerability ranking and for each one of the four themes. Using this ranking system, each census tract and county can be used for mapping analyses of relative vulnerability, ranging from 0 to 1, with higher values indicative of increasing vulnerability. In our analysis, we classified the ranking of overall SVI among US counties into four different quartiles as follows: (0 – 0.25 as 1^st^ quartile and least vulnerable, >0.25 – 0.50 as 2^nd^ quartile, >0.50 – 0.75 as 3^rd^ quartile, and >0.75 – 1.00 as 4^th^ quartile and most vulnerable).

We estimated AAMR per 100,000 person-years with 95% confidence intervals for all US residents from 1999 to 2020 that included ischemic heart diseases as the underlying cause of death. We additionally obtained AAMR within each subgroup component at a racial, gender, and geographic level. Data is available in **Table S2**. We aggregated counties into the four SVI quartiles and compared AAMR from 2014 to 2018 among the most vulnerable quartile and the least vulnerable quartile (**Table S5**). Risk ratio (RR) and associated 95% confidence intervals (CIs) were estimated comparing the AAMR between the 1st and 4th quartiles by Poisson univariable regression; 95% CIs that did not cross 1.0 were considered statistically significant.

**Supplemental Table 1. Social Vulnerability Index.** Characterization of the 15 social factors used to assess social vulnerability.

| **Socioeconomic Status** | **Household Composition and Disability** | **Minority Status and Language** | **Housing Type and Transportation** |
| --- | --- | --- | --- |
| Unemployed | Aged 65 or Older | Minority | Multi-Unit Structures |
| Below Poverty | Aged 17 or Younger | Speaks English "Less than Well" | Mobile Homes |
| Income | Older than Age 5 with a Disability |  | Crowding |
| No High School Diploma | Single-Parent Households |  | Group Quarters |
|  |  |  | No Vehicle |

**Supplemental Table 2**. **Cumulative Mortality Data**. Table depicting yearly overall death rate, total population, crude and age-adjusted mortality rates with associated 95% confidence intervals. Cumulative age-adjusted mortality rates from 1999 to 2020 are also depicted, along with their average annual percentage change.

| **Groups** | **Year** | **Deaths** | **Population** | **CMR (95% CI)** | **AAMR (95% CI)** | **Cumulative AAMR (95% CI)** | **AAPC (95% CI)** |
| --- | --- | --- | --- | --- | --- | --- | --- |
| **All** | 1999 | 529659 | 279040168 | 189.81 (189.3-190.33) | 194.6 (194.07-195.12) | 123.04 (122.96– 123.12) | -3.6% (-3.9 – -3.2) |
|  | 2000 | 515204 | 281421906 | 183.07 (182.57-183.57) | 186.76 (186.25-187.27) |  |  |
|  | 2001 | 502189 | 284968955 | 176.23 (175.74-176.71) | 178.96 (178.46-179.45) |  |  |
|  | 2002 | 494382 | 287625193 | 171.88 (171.4-172.36) | 173.5 (173.01-173.98) |  |  |
|  | 2003 | 480028 | 290107933 | 165.47 (165-165.93) | 165.55 (165.08-166.02) |  |  |
|  | 2004 | 451326 | 292805298 | 154.14 (153.69-154.59) | 153.24 (152.79-153.69) |  |  |
|  | 2005 | 445687 | 295516599 | 150.82 (150.37-151.26) | 148.15 (147.71-148.59) |  |  |
|  | 2006 | 425425 | 298379912 | 142.58 (142.15-143.01) | 138.33 (137.91-138.75) |  |  |
|  | 2007 | 406351 | 301231207 | 134.9 (134.48-135.31) | 129.24 (128.84-129.64) |  |  |
|  | 2008 | 405309 | 304093966 | 133.28 (132.87-133.69) | 126.14 (125.75-126.53) |  |  |
|  | 2009 | 386324 | 306771529 | 125.93 (125.54-126.33) | 117.72 (117.34-118.09) |  |  |
|  | 2010 | 379559 | 308745538 | 122.94 (122.54-123.33) | 113.65 (113.28-114.01) |  |  |
|  | 2011 | 375295 | 311591917 | 120.44 (120.06-120.83) | 109.18 (108.83-109.53) |  |  |
|  | 2012 | 371469 | 313914040 | 118.33 (117.95-118.72) | 105.44 (105.1-105.78) |  |  |
|  | 2013 | 370213 | 316128839 | 117.11 (116.73-117.49) | 102.65 (102.31-102.98) |  |  |
|  | 2014 | 364593 | 318857056 | 114.34 (113.97-114.71) | 98.83 (98.5-99.15) |  |  |
|  | 2015 | 366801 | 321418820 | 114.12 (113.75-114.49) | 97.24 (96.92-97.55) |  |  |
|  | 2016 | 363452 | 323127513 | 112.48 (112.11-112.85) | 94.33 (94.02-94.65) |  |  |
|  | 2017 | 365914 | 325719178 | 112.34 (111.98-112.7) | 92.88 (92.58-93.19) |  |  |
|  | 2018 | 365744 | 327167434 | 111.79 (111.43-112.15) | 90.85 (90.56-91.15) |  |  |
|  | 2019 | 360900 | 328239523 | 109.95 (109.59-110.31) | 87.97 (87.67-88.26) |  |  |
|  | 2020 | 382820 | 329484123 | 116.19 (115.82-116.56) | 91.8 (91.5-92.09) |  |  |
| **Female** | 1999 | 262391 | 142237295 | 184.47 (183.77-185.18) | 152.93 (152.34-153.52) | 93.16 (93.07– 93.25) | -4.1% (-4.5 – -3.7) |
|  | 2000 | 254630 | 143368343 | 177.61 (176.92-178.3) | 146.47 (145.9-147.04) |  |  |
|  | 2001 | 248184 | 145077463 | 171.07 (170.4-171.74) | 140.91 (140.35-141.46) |  |  |
|  | 2002 | 241622 | 146394634 | 165.05 (164.39-165.71) | 135.67 (135.13-136.21) |  |  |
|  | 2003 | 233686 | 147679036 | 158.24 (157.6-158.88) | 129.43 (128.9-129.96) |  |  |
|  | 2004 | 217788 | 148977286 | 146.19 (145.57-146.8) | 119.4 (118.9-119.9) |  |  |
|  | 2005 | 213572 | 150319521 | 142.08 (141.48-142.68) | 115 (114.51-115.49) |  |  |
|  | 2006 | 200915 | 151732647 | 132.41 (131.83-132.99) | 106.26 (105.79-106.73) |  |  |
|  | 2007 | 190301 | 153166353 | 124.24 (123.69-124.8) | 98.76 (98.31-99.21) |  |  |
|  | 2008 | 189061 | 154604015 | 122.29 (121.74-122.84) | 96.34 (95.9-96.78) |  |  |
|  | 2009 | 176255 | 155964075 | 113.01 (112.48-113.54) | 88.4 (87.98-88.81) |  |  |
|  | 2010 | 171979 | 156964212 | 109.57 (109.05-110.08) | 84.85 (84.45-85.26) |  |  |
|  | 2011 | 168387 | 158301098 | 106.37 (105.86-106.88) | 81.02 (80.63-81.41) |  |  |
|  | 2012 | 164784 | 159421973 | 103.36 (102.86-103.86) | 77.8 (77.41-78.18) |  |  |
|  | 2013 | 161698 | 160477237 | 100.76 (100.27-101.25) | 74.93 (74.56-75.3) |  |  |
|  | 2014 | 157181 | 161920569 | 97.07 (96.59-97.55) | 71.65 (71.29-72.01) |  |  |
|  | 2015 | 157503 | 163189523 | 96.52 (96.04-96.99) | 70.51 (70.16-70.87) |  |  |
|  | 2016 | 153296 | 164048590 | 93.45 (92.98-93.91) | 67.55 (67.2-67.89) |  |  |
|  | 2017 | 152619 | 165311059 | 92.32 (91.86-92.79) | 65.96 (65.62-66.29) |  |  |
|  | 2018 | 150712 | 166038755 | 90.77 (90.31-91.23) | 64 (63.67-64.33) |  |  |
|  | 2019 | 147536 | 166582199 | 88.57 (88.11-89.02) | 61.7 (61.38-62.02) |  |  |
|  | 2020 | 154933 | 167227921 | 92.65 (92.19-93.11) | 64.06 (63.73-64.38) |  |  |
| **Male** | 1999 | 267268 | 136802873 | 195.37 (194.63-196.11) | 251.22 (250.24-252.19) | 161.51 (161.37– 161.66) | -3.5% (-3.7 – -3.2) |
|  | 2000 | 260574 | 138053563 | 188.75 (188.02-189.47) | 241.38 (240.44-242.33) |  |  |
|  | 2001 | 254005 | 139891492 | 181.57 (180.87-182.28) | 230.21 (229.3-231.13) |  |  |
|  | 2002 | 252760 | 141230559 | 178.97 (178.27-179.67) | 224.66 (223.77-225.56) |  |  |
|  | 2003 | 246342 | 142428897 | 172.96 (172.27-173.64) | 213.9 (213.04-214.76) |  |  |
|  | 2004 | 233538 | 143828012 | 162.37 (161.71-163.03) | 198.42 (197.6-199.24) |  |  |
|  | 2005 | 232115 | 145197078 | 159.86 (159.21-160.51) | 192.34 (191.54-193.14) |  |  |
|  | 2006 | 224510 | 146647265 | 153.1 (152.46-153.73) | 180.73 (179.97-181.49) |  |  |
|  | 2007 | 216050 | 148064854 | 145.92 (145.3-146.53) | 169.24 (168.52-169.97) |  |  |
|  | 2008 | 216248 | 149489951 | 144.66 (144.05-145.27) | 165.14 (164.43-165.84) |  |  |
|  | 2009 | 210069 | 150807454 | 139.3 (138.7-139.89) | 156.22 (155.54-156.9) |  |  |
|  | 2010 | 207580 | 151781326 | 136.76 (136.17-137.35) | 151.28 (150.61-151.94) |  |  |
|  | 2011 | 206908 | 153290819 | 134.98 (134.4-135.56) | 145.58 (144.94-146.22) |  |  |
|  | 2012 | 206685 | 154492067 | 133.78 (133.21-134.36) | 141.07 (140.45-141.69) |  |  |
|  | 2013 | 208515 | 155651602 | 133.96 (133.39-134.54) | 138.17 (137.57-138.78) |  |  |
|  | 2014 | 207412 | 156936487 | 132.16 (131.59-132.73) | 133.48 (132.9-134.06) |  |  |
|  | 2015 | 209298 | 158229297 | 132.28 (131.71-132.84) | 131.16 (130.59-131.73) |  |  |
|  | 2016 | 210156 | 159078923 | 132.11 (131.54-132.67) | 128.31 (127.75-128.87) |  |  |
|  | 2017 | 213295 | 160408119 | 132.97 (132.41-133.53) | 126.8 (126.25-127.35) |  |  |
|  | 2018 | 215032 | 161128679 | 133.45 (132.89-134.02) | 124.53 (123.99-125.06) |  |  |
|  | 2019 | 213364 | 161657324 | 131.99 (131.43-132.55) | 120.86 (120.33-121.38) |  |  |
|  | 2020 | 227887 | 162256202 | 140.45 (139.87-141.03) | 126.19 (125.67-126.72) |  |  |
| **Hispanic** | 1999 | 19936 | 33937795 | 58.74 (57.93-59.56) | 162.15 (159.81-164.5) | 92.94 (92.67–93.21) | -3.7% (-4.2 – -3.1) |
|  | 2000 | 19744 | 35305818 | 55.92 (55.14-56.7) | 153.21 (150.99-155.44) |  |  |
|  | 2001 | 20664 | 37144096 | 55.63 (54.87-56.39) | 151.07 (148.92-153.21) |  |  |
|  | 2002 | 20941 | 38617620 | 54.23 (53.49-54.96) | 144.75 (142.7-146.79) |  |  |
|  | 2003 | 20783 | 40049429 | 51.89 (51.19-52.6) | 136.81 (134.87-138.75) |  |  |
|  | 2004 | 20482 | 41501375 | 49.35+AM7 (48.68-50.03) | 127.39 (125.57-129.21) |  |  |
|  | 2005 | 21774 | 43023614 | 50.61 (49.94-51.28) | 127.85 (126.08-129.62) |  |  |
|  | 2006 | 20939 | 44606305 | 46.94 (46.31-47.58) | 116.41 (114.77-118.05) |  |  |
|  | 2007 | 20452 | 46196853 | 44.27 (43.66-44.88) | 107.54 (106.01-109.08) |  |  |
|  | 2008 | 20261 | 47793785 | 42.39 (41.81-42.98) | 100.82 (99.38-102.26) |  |  |
|  | 2009 | 20228 | 49327489 | 41.01 (40.44-41.57) | 94.7 (93.35-96.06) |  |  |
|  | 2010 | 20494 | 50477594 | 40.6 (40.04-41.16) | 92.28 (90.97-93.59) |  |  |
|  | 2011 | 20326 | 52045277 | 39.05 (38.52-39.59) | 84.17 (82.97-85.37) |  |  |
|  | 2012 | 20751 | 53027708 | 39.13 (38.6-39.66) | 81.05 (79.91-82.19) |  |  |
|  | 2013 | 21788 | 54071370 | 40.29 (39.76-40.83) | 80.35 (79.25-81.45) |  |  |
|  | 2014 | 21871 | 55387539 | 39.49 (38.96-40.01) | 75.27 (74.24-76.3) |  |  |
|  | 2015 | 23055 | 56592793 | 40.74 (40.21-41.26) | 74.55 (73.56-75.54) |  |  |
|  | 2016 | 23574 | 57470287 | 41.02 (40.5-41.54) | 72.68 (71.72-73.63) |  |  |
|  | 2017 | 24236 | 58946729 | 41.12 (40.6-41.63) | 70.69 (69.78-71.61) |  |  |
|  | 2018 | 24860 | 59871746 | 41.52 (41.01-42.04) | 69.12 (68.23-70) |  |  |
|  | 2019 | 25348 | 60572237 | 41.85 (41.33-42.36) | 67.77 (66.91-68.63) |  |  |
|  | 2020 | 29623 | 61312879 | 48.31 (47.76-48.86) | 75.57 (74.69-76.46) |  |  |
| **Non-Hispanic** | 1999 | 507700 | 245102373 | 207.14 (206.57-207.71) | 195.69 (195.15-196.23) | 125.20 (125.12– 125.29) | -3.5% (-3.9 – 3.1) |
|  | 2000 | 493541 | 246116088 | 200.53 (199.97-201.09) | 188.01 (187.48-188.53) |  |  |
|  | 2001 | 479842 | 247824859 | 193.62 (193.07-194.17) | 180.17 (179.66-180.68) |  |  |
|  | 2002 | 471655 | 249007573 | 189.41 (188.87-189.95) | 174.74 (174.24-175.24) |  |  |
|  | 2003 | 457794 | 250058504 | 183.07 (182.54-183.61) | 167 (166.51-167.48) |  |  |
|  | 2004 | 429713 | 251303923 | 170.99 (170.48-171.5) | 154.67 (154.2-155.13) |  |  |
|  | 2005 | 423007 | 252492985 | 167.53 (167.03-168.04) | 149.4 (148.95-149.85) |  |  |
|  | 2006 | 403588 | 253773607 | 159.03 (158.54-159.53) | 139.79 (139.36-140.22) |  |  |
|  | 2007 | 385210 | 255034354 | 151.04 (150.57-151.52) | 130.83 (130.42-131.25) |  |  |
|  | 2008 | 384127 | 256300181 | 149.87 (149.4-150.35) | 127.95 (127.54-128.35) |  |  |
|  | 2009 | 365119 | 257444040 | 141.82 (141.36-142.28) | 119.39 (119-119.78) |  |  |
|  | 2010 | 357969 | 258267944 | 138.6 (138.15-139.06) | 115.24 (114.85-115.62) |  |  |
|  | 2011 | 353962 | 259546640 | 136.38 (135.93-136.83) | 111.11 (110.74-111.48) |  |  |
|  | 2012 | 349281 | 260886332 | 133.88 (133.44-134.33) | 107.31 (106.95-107.67) |  |  |
|  | 2013 | 347126 | 262057469 | 132.46 (132.02-132.9) | 104.49 (104.14-104.84) |  |  |
|  | 2014 | 341091 | 263469517 | 129.46 (129.03-129.9) | 100.74 (100.4-101.09) |  |  |
|  | 2015 | 341869 | 264826027 | 129.09 (128.66-129.52) | 99.06 (98.72-99.39) |  |  |
|  | 2016 | 338346 | 265657226 | 127.36 (126.93-127.79) | 96.31 (95.98-96.64) |  |  |
|  | 2017 | 340097 | 266772449 | 127.49 (127.06-127.91) | 94.93 (94.6-95.25) |  |  |
|  | 2018 | 339418 | 267295688 | 126.98 (126.56-127.41) | 92.96 (92.64-93.28) |  |  |
|  | 2019 | 334369 | 267667286 | 124.92 (124.5-125.34) | 90.05 (89.74-90.36) |  |  |
|  | 2020 | 351843 | 268171244 | 131.2 (130.77-131.63) | 93.54 (93.23-93.86) |  |  |
| **White** | 1999 | 469149 | 228687790 | 205.15 (204.56-205.74) | 193.4 (192.85-193.96) | 123.34 (123.25– 123.43) | -3.6% (-4.0 – -3.2) |
|  | 2000 | 455400 | 230085762 | 197.93 (197.35-198.5) | 185.56 (185.03-186.1) |  |  |
|  | 2001 | 442536 | 232192666 | 190.59 (190.03-191.15) | 177.59 (177.07-178.12) |  |  |
|  | 2002 | 435170 | 233720535 | 186.19 (185.64-186.75) | 172.29 (171.77-172.8) |  |  |
|  | 2003 | 421482 | 235125072 | 179.26 (178.72-179.8) | 164.2 (163.7-164.69) |  |  |
|  | 2004 | 395705 | 236670034 | 167.2 (166.68-167.72) | 152.12 (151.64-152.59) |  |  |
|  | 2005 | 390421 | 238187095 | 163.91 (163.4-164.43) | 147.29 (146.83-147.76) |  |  |
|  | 2006 | 371445 | 239805271 | 154.89 (154.4-155.39) | 137.38 (136.93-137.82) |  |  |
|  | 2007 | 354481 | 241390828 | 146.85 (146.37-147.33) | 128.52 (128.09-128.94) |  |  |
|  | 2008 | 353839 | 242966379 | 145.63 (145.15-146.11) | 125.81 (125.39-126.23) |  |  |
|  | 2009 | 336238 | 244388833 | 137.58 (137.12-138.05) | 117.39 (116.99-117.79) |  |  |
|  | 2010 | 330277 | 245423340 | 134.57 (134.12-135.03) | 113.53 (113.14-113.92) |  |  |
|  | 2011 | 326101 | 247094331 | 131.97 (131.52-132.43) | 109.31 (108.93-109.69) |  |  |
|  | 2012 | 321773 | 248224553 | 129.63 (129.18-130.08) | 105.63 (105.26-106) |  |  |
|  | 2013 | 319783 | 249344498 | 128.25 (127.8-128.69) | 102.9 (102.54-103.26) |  |  |
|  | 2014 | 314360 | 250630467 | 125.43 (124.99-125.87) | 99.29 (98.94-99.64) |  |  |
|  | 2015 | 315829 | 251875054 | 125.39 (124.95-125.83) | 97.96 (97.61-98.31) |  |  |
|  | 2016 | 311224 | 252702814 | 123.16 (122.73-123.59) | 94.8 (94.47-95.14) |  |  |
|  | 2017 | 312837 | 253935650 | 123.2 (122.76-123.63) | 93.53 (93.2-93.87) |  |  |
|  | 2018 | 311617 | 254564236 | 122.41 (121.98-122.84) | 91.41 (91.09-91.74) |  |  |
|  | 2019 | 306947 | 255040203 | 120.35 (119.93-120.78) | 88.65 (88.34-88.97) |  |  |
|  | 2020 | 320847 | 254989656 | 125.83 (125.39-126.26) | 91.54 (91.22-91.86) |  |  |
| **Black** | 1999 | 51995 | 36173121 | 143.74 (142.5-144.97) | 224.92 (222.97-226.88) | 141.49 (141.20– 141.78) | -3.4% (-3.7 – -3.1) |
|  | 2000 | 51265 | 36594309 | 140.09 (138.88-141.3) | 218.31 (216.4-220.23) |  |  |
|  | 2001 | 50933 | 37249775 | 136.73 (135.55-137.92) | 212.92 (211.04-214.79) |  |  |
|  | 2002 | 50174 | 37748424 | 132.92 (131.75-134.08) | 206.35 (204.51-208.18) |  |  |
|  | 2003 | 49274 | 38209741 | 128.96 (127.82-130.1) | 198.58 (196.8-200.37) |  |  |
|  | 2004 | 46695 | 38738798 | 120.54 (119.44-121.63) | 183.77 (182.07-185.47) |  |  |
|  | 2005 | 46027 | 39280731 | 117.17 (116.1-118.24) | 175.68 (174.04-177.32) |  |  |
|  | 2006 | 44530 | 39857107 | 111.72 (110.69-112.76) | 165.36 (163.79-166.94) |  |  |
|  | 2007 | 42679 | 40451108 | 105.51 (104.51-106.51) | 153.99 (152.49-155.49) |  |  |
|  | 2008 | 41898 | 41048959 | 102.07 (101.09-103.05) | 146.77 (145.33-148.21) |  |  |
|  | 2009 | 40521 | 41632450 | 97.33 (96.38-98.28) | 137.39 (136.01-138.76) |  |  |
|  | 2010 | 39630 | 42065334 | 94.21 (93.28-95.14) | 131.21 (129.88-132.54) |  |  |
|  | 2011 | 39453 | 42678815 | 92.44 (91.53-93.35) | 125.34 (124.07-126.62) |  |  |
|  | 2012 | 39675 | 43203092 | 91.83 (90.93-92.74) | 121.29 (120.06-122.52) |  |  |
|  | 2013 | 39784 | 43696271 | 91.05 (90.15-91.94) | 117.46 (116.27-118.65) |  |  |
|  | 2014 | 39604 | 44309394 | 89.38 (88.5-90.26) | 112.78 (111.63-113.92) |  |  |
|  | 2015 | 39730 | 44863196 | 88.56 (87.69-89.43) | 108.92 (107.81-110.02) |  |  |
|  | 2016 | 40814 | 45307020 | 90.08 (89.21-90.96) | 108.27 (107.19-109.35) |  |  |
|  | 2017 | 40916 | 45883808 | 89.17 (88.31-90.04) | 104.74 (103.69-105.78) |  |  |
|  | 2018 | 41575 | 46262846 | 89.87 (89-90.73) | 103.23 (102.21-104.25) |  |  |
|  | 2019 | 41418 | 46599393 | 88.88 (88.02-89.74) | 100.04 (99.05-101.03) |  |  |
|  | 2020 | 47542 | 47181245 | 100.76 (99.86-101.67) | 111.49 (110.46-112.52) |  |  |
| **Asian/Pacific Islander** | 1999 | 6795 | 11346496 | 59.89 (58.46-61.31) | 117.29 (114.36-120.22) | 66.88 (66.57–67.20) | -3.6% (-4.0 – -3.2) |
|  | 2000 | 6799 | 11757685 | 57.83 (56.45-59.2) | 109.6 (106.88-112.32) |  |  |
|  | 2001 | 7010 | 12429181 | 56.4 (55.08-57.72) | 104.13 (101.59-106.67) |  |  |
|  | 2002 | 7261 | 12953763 | 56.05 (54.76-57.34) | 101.91 (99.47-104.34) |  |  |
|  | 2003 | 7367 | 13461109 | 54.73 (53.48-55.98) | 96.68 (94.39-98.98) |  |  |
|  | 2004 | 7083 | 13967014 | 50.71 (49.53-51.89) | 88.31 (86.18-90.44) |  |  |
|  | 2005 | 7451 | 14494417 | 51.41 (50.24-52.57) | 85.55 (83.54-87.56) |  |  |
|  | 2006 | 7570 | 15029851 | 50.37 (49.23-51.5) | 82.24 (80.32-84.15) |  |  |
|  | 2007 | 7414 | 15559373 | 47.65 (46.57-48.73) | 75.87 (74.09-77.65) |  |  |
|  | 2008 | 7833 | 16094699 | 48.67 (47.59-49.75) | 75.89 (74.16-77.62) |  |  |
|  | 2009 | 7752 | 16609031 | 46.67 (45.63-47.71) | 70.7 (69.09-72.32) |  |  |
|  | 2010 | 7821 | 16993326 | 46.02 (45-47.04) | 68.68 (67.12-70.24) |  |  |
|  | 2011 | 7828 | 17471358 | 44.8 (43.81-45.8) | 62.72 (61.3-64.14) |  |  |
|  | 2012 | 8071 | 18087959 | 44.62 (43.65-45.59) | 60.53 (59.18-61.87) |  |  |
|  | 2013 | 8610 | 18630136 | 46.22 (45.24-47.19) | 59.95 (58.66-61.24) |  |  |
|  | 2014 | 8512 | 19398214 | 43.88 (42.95-44.81) | 55.15 (53.95-56.34) |  |  |
|  | 2015 | 9094 | 20102717 | 45.24 (44.31-46.17) | 54.95 (53.8-56.1) |  |  |
|  | 2016 | 9247 | 20487524 | 45.13 (44.21-46.05) | 53.03 (51.93-54.12) |  |  |
|  | 2017 | 10008 | 21210673 | 47.18 (46.26-48.11) | 53.87 (52.8-54.94) |  |  |
|  | 2018 | 10351 | 21601064 | 47.92 (47-48.84) | 53 (51.96-54.03) |  |  |
|  | 2019 | 10404 | 21814724 | 47.69 (46.78-48.61) | 50.67 (49.68-51.65) |  |  |
|  | 2020 | 12128 | 22413737 | 54.11 (53.15-55.07) | 56.07 (55.06-57.07) |  |  |
| **American Indian/Alaska Native** | 1999 | 1720 | 2832761 | 60.72 (57.85-63.59) | 143.26 (136.03-150.48) | 85.10 (84.24–85.96) | -3.7% (-4.8 – -2.6) |
|  | 2000 | 1740 | 2984150 | 58.31 (55.57-61.05) | 129.1 (122.67-135.53) |  |  |
|  | 2001 | 1710 | 3097333 | 55.21 (52.59-57.83) | 119.59 (113.54-125.64) |  |  |
|  | 2002 | 1777 | 3202471 | 55.49 (52.91-58.07) | 122.8 (116.7-128.91) |  |  |
|  | 2003 | 1905 | 3312011 | 57.52 (54.93-60.1) | 124.39 (118.37-130.4) |  |  |
|  | 2004 | 1843 | 3429452 | 53.74 (51.29-56.19) | 118.77 (112.93-124.62) |  |  |
|  | 2005 | 1788 | 3554356 | 50.3 (47.97-52.64) | 106.13 (100.8-111.45) |  |  |
|  | 2006 | 1880 | 3687683 | 50.98 (48.68-53.29) | 107.24 (102.01-112.47) |  |  |
|  | 2007 | 1777 | 3829898 | 46.4 (44.24-48.56) | 95.92 (91.09-100.74) |  |  |
|  | 2008 | 1739 | 3983929 | 43.65 (41.6-45.7) | 88 (83.51-92.49) |  |  |
|  | 2009 | 1813 | 4141215 | 43.78 (41.76-45.79) | 86.47 (82.15-90.78) |  |  |
|  | 2010 | 1831 | 4263538 | 42.95 (40.98-44.91) | 84.94 (80.73-89.16) |  |  |
|  | 2011 | 1913 | 4347413 | 44 (42.03-45.98) | 81.41 (77.48-85.34) |  |  |
|  | 2012 | 1950 | 4398436 | 44.33 (42.37-46.3) | 79.15 (75.38-82.93) |  |  |
|  | 2013 | 2036 | 4457934 | 45.67 (43.69-47.66) | 78.19 (74.56-81.83) |  |  |
|  | 2014 | 2117 | 4518981 | 46.85 (44.85-48.84) | 76.37 (72.9-79.84) |  |  |
|  | 2015 | 2148 | 4577853 | 46.92 (44.94-48.91) | 73.36 (70.07-76.66) |  |  |
|  | 2016 | 2167 | 4630155 | 46.8 (44.83-48.77) | 71.31 (68.14-74.49) |  |  |
|  | 2017 | 2153 | 4689047 | 45.92 (43.98-47.86) | 67.8 (64.78-70.82) |  |  |
|  | 2018 | 2201 | 4739288 | 46.44 (44.5-48.38) | 64.7 (61.86-67.53) |  |  |
|  | 2019 | 2131 | 4785203 | 44.53 (42.64-46.42) | 60.12 (57.45-62.79) |  |  |
|  | 2020 | 2303 | 4899485 | 47 (45.09-48.92) | 62.26 (59.62-64.91) |  |  |
| **Metropolitan** | 1999 | 426797 | 234421194 | 182.06 (181.52-182.61) | 193.96 (193.38-194.54) | 120.42 (120.33– 120.51) | -3.7% (-4.1 – -3.3) |
|  | 2000 | 414584 | 236637796 | 175.2 (174.66-175.73) | 185.71 (185.15-186.28) |  |  |
|  | 2001 | 405930 | 240128266 | 169.05 (168.53-169.57) | 178.39 (177.85-178.94) |  |  |
|  | 2002 | 398852 | 242665792 | 164.36 (163.85-164.87) | 172.35 (171.81-172.88) |  |  |
|  | 2003 | 387723 | 244993933 | 158.26 (157.76-158.76) | 164.3 (163.79-164.82) |  |  |
|  | 2004 | 365014 | 247510633 | 147.47 (147-147.95) | 152.06 (151.57-152.55) |  |  |
|  | 2005 | 360129 | 250039935 | 144.03 (143.56-144.5) | 146.56 (146.08-147.04) |  |  |
|  | 2006 | 344140 | 252641624 | 136.22 (135.76-136.67) | 136.84 (136.38-137.3) |  |  |
|  | 2007 | 328169 | 255300617 | 128.54 (128.1-128.98) | 127.43 (127-127.87) |  |  |
|  | 2008 | 326539 | 257996520 | 126.57 (126.13-127) | 123.79 (123.36-124.22) |  |  |
|  | 2009 | 311227 | 260573371 | 119.44 (119.02-119.86) | 115.32 (114.91-115.73) |  |  |
|  | 2010 | 305476 | 262452132 | 116.39 (115.98-116.81) | 111.07 (110.68-111.47) |  |  |
|  | 2011 | 302422 | 265270604 | 114.01 (113.6-114.41) | 106.6 (106.22-106.98) |  |  |
|  | 2012 | 298938 | 267664440 | 111.68 (111.28-112.08) | 102.6 (102.23-102.97) |  |  |
|  | 2013 | 297831 | 269911242 | 110.34 (109.95-110.74) | 99.69 (99.33-100.05) |  |  |
|  | 2014 | 292964 | 272667942 | 107.44 (107.05-107.83) | 95.66 (95.31-96.01) |  |  |
|  | 2015 | 294590 | 275252217 | 107.03 (106.64-107.41) | 93.89 (93.54-94.23) |  |  |
|  | 2016 | 292626 | 277016929 | 105.63 (105.25-106.02) | 91.17 (90.84-91.51) |  |  |
|  | 2017 | 294923 | 279636439 | 105.47 (105.09-105.85) | 89.73 (89.4-90.06) |  |  |
|  | 2018 | 295281 | 281067210 | 105.06 (104.68-105.44) | 87.8 (87.48-88.12) |  |  |
|  | 2019 | 291354 | 282176462 | 103.25 (102.88-103.63) | 84.95 (84.64-85.26) |  |  |
|  | 2020 | 310052 | 283450351 | 109.38 (109-109.77) | 88.79 (88.48-89.11) |  |  |
| **Non-metropolitan** | 1999 | 102862 | 44618974 | 230.53 (229.13-231.94) | 198.16 (196.94-199.37) | 136.17 (135.97– 136.37) | -3.1% (-3.3 – -2.8) |
|  | 2000 | 100620 | 44784110 | 224.68 (223.29-226.07) | 192.15 (190.96-193.34) |  |  |
|  | 2001 | 96259 | 44840689 | 214.67 (213.31-216.03) | 182.47 (181.31-183.62) |  |  |
|  | 2002 | 95530 | 44959401 | 212.48 (211.13-213.83) | 179.72 (178.57-180.86) |  |  |
|  | 2003 | 92305 | 45114000 | 204.6 (203.28-205.92) | 171.95 (170.83-173.06) |  |  |
|  | 2004 | 86312 | 45294665 | 190.56 (189.29-191.83) | 159.36 (158.29-160.42) |  |  |
|  | 2005 | 85558 | 45476664 | 188.14 (186.88-189.4) | 155.96 (154.91-157.01) |  |  |
|  | 2006 | 81285 | 45738288 | 177.72 (176.5-178.94) | 145.84 (144.83-146.84) |  |  |
|  | 2007 | 78182 | 45930590 | 170.22 (169.02-171.41) | 138.21 (137.24-139.18) |  |  |
|  | 2008 | 78770 | 46097446 | 170.88 (169.68-172.07) | 137.44 (136.48-138.41) |  |  |
|  | 2009 | 75097 | 46198158 | 162.55 (161.39-163.72) | 129.4 (128.47-130.34) |  |  |
|  | 2010 | 74083 | 46293406 | 160.03 (158.88-161.18) | 126.12 (125.21-127.04) |  |  |
|  | 2011 | 72873 | 46321313 | 157.32 (156.18-158.46) | 122.11 (121.22-123.01) |  |  |
|  | 2012 | 72531 | 46249600 | 156.83 (155.68-157.97) | 119.78 (118.9-120.66) |  |  |
|  | 2013 | 72382 | 46217597 | 156.61 (155.47-157.75) | 117.7 (116.83-118.57) |  |  |
|  | 2014 | 71629 | 46189114 | 155.08 (153.94-156.21) | 115.12 (114.27-115.98) |  |  |
|  | 2015 | 72211 | 46166603 | 156.41 (155.27-157.55) | 114.6 (113.74-115.45) |  |  |
|  | 2016 | 70826 | 46110584 | 153.6 (152.47-154.73) | 110.95 (110.11-111.78) |  |  |
|  | 2017 | 70991 | 46082739 | 154.05 (152.92-155.18) | 109.42 (108.6-110.24) |  |  |
|  | 2018 | 70463 | 46100224 | 152.85 (151.72-153.98) | 106.95 (106.14-107.76) |  |  |
|  | 2019 | 69546 | 46063061 | 150.98 (149.86-152.1) | 104.12 (103.33-104.91) |  |  |
|  | 2020 | 72768 | 46024426 | 158.11 (156.96-159.26) | 108.11 (107.3-108.91) |  |  |
| **Northeast** | 1999 | 120668 | 53343775 | 226.21 (224.93-227.48) | 207.88 (206.71-209.05) | 132.96 (132.77– 133.15) | -3.6% (-4.0 – -3.1) |
|  | 2000 | 117766 | 53594378 | 219.74 (218.48-220.99) | 200.2 (199.06-201.34) |  |  |
|  | 2001 | 114247 | 53915522 | 211.9 (210.67-213.13) | 191.61 (190.5-192.72) |  |  |
|  | 2002 | 113076 | 54143915 | 208.84 (207.63-210.06) | 187.2 (186.11-188.3) |  |  |
|  | 2003 | 109494 | 54334453 | 201.52 (200.32-202.71) | 178.67 (177.61-179.73) |  |  |
|  | 2004 | 102291 | 54423533 | 187.95 (186.8-189.11) | 165.28 (164.27-166.3) |  |  |
|  | 2005 | 100252 | 54451230 | 184.11 (182.97-185.25) | 159.59 (158.6-160.58) |  |  |
|  | 2006 | 94256 | 54522659 | 172.87 (171.77-173.98) | 147.85 (146.9-148.8) |  |  |
|  | 2007 | 91211 | 54653362 | 166.89 (165.81-167.97) | 140.83 (139.91-141.75) |  |  |
|  | 2008 | 90788 | 54875926 | 165.44 (164.37-166.52) | 137.87 (136.97-138.77) |  |  |
|  | 2009 | 85537 | 55133101 | 155.15 (154.11-156.19) | 128.17 (127.3-129.04) |  |  |
|  | 2010 | 81918 | 55317240 | 148.09 (147.07-149.1) | 121.02 (120.18-121.85) |  |  |
|  | 2011 | 80236 | 55521598 | 144.51 (143.51-145.51) | 116.25 (115.43-117.06) |  |  |
|  | 2012 | 78166 | 55761091 | 140.18 (139.2-141.16) | 111.17 (110.38-111.96) |  |  |
|  | 2013 | 77568 | 55943073 | 138.66 (137.68-139.63) | 108.76 (107.98-109.54) |  |  |
|  | 2014 | 75029 | 56152333 | 133.62 (132.66-134.57) | 103.81 (103.05-104.56) |  |  |
|  | 2015 | 75158 | 56283891 | 133.53 (132.58-134.49) | 102.63 (101.88-103.38) |  |  |
|  | 2016 | 73678 | 56209510 | 131.08 (130.13-132.02) | 99.65 (98.91-100.38) |  |  |
|  | 2017 | 73626 | 56470581 | 130.38 (129.44-131.32) | 96.9 (96.19-97.61) |  |  |
|  | 2018 | 73767 | 56111079 | 131.47 (130.52-132.41) | 96.1 (95.4-96.81) |  |  |
|  | 2019 | 71708 | 55982803 | 128.09 (127.15-129.03) | 92.34 (91.65-93.03) |  |  |
|  | 2020 | 76366 | 55849869 | 136.73 (135.76-137.7) | 97.75 (97.05-98.45) |  |  |
| **Midwest** | 1999 | 127255 | 64100061 | 198.53 (197.43-199.62) | 194.07 (193-195.13) | 124.68 (124.51– 124.85) | -3.5% (-3.8 – -3.1) |
|  | 2000 | 122773 | 64392776 | 190.66 (189.6-191.73) | 185.62 (184.58-186.66) |  |  |
|  | 2001 | 118194 | 64776531 | 182.46 (181.42-183.5) | 176.44 (175.43-177.44) |  |  |
|  | 2002 | 115360 | 65018293 | 177.43 (176.4-178.45) | 170.18 (169.19-171.16) |  |  |
|  | 2003 | 110935 | 65276954 | 169.95 (168.95-170.95) | 161.34 (160.39-162.29) |  |  |
|  | 2004 | 104042 | 65532305 | 158.76 (157.8-159.73) | 149.6 (148.69-150.51) |  |  |
|  | 2005 | 102744 | 65751872 | 156.26 (155.3-157.22) | 145.49 (144.6-146.39) |  |  |
|  | 2006 | 98785 | 66028555 | 149.61 (148.68-150.54) | 137.45 (136.59-138.31) |  |  |
|  | 2007 | 93769 | 66293689 | 141.44 (140.54-142.35) | 128.13 (127.3-128.95) |  |  |
|  | 2008 | 94637 | 66523935 | 142.26 (141.35-143.17) | 127.21 (126.39-128.02) |  |  |
|  | 2009 | 88414 | 66748437 | 132.46 (131.59-133.33) | 117.23 (116.46-118.01) |  |  |
|  | 2010 | 87862 | 66927001 | 131.28 (130.41-132.15) | 114.75 (113.99-115.52) |  |  |
|  | 2011 | 86945 | 67158835 | 129.46 (128.6-130.32) | 111.22 (110.47-111.97) |  |  |
|  | 2012 | 86263 | 67316297 | 128.15 (127.29-129) | 108.49 (107.76-109.22) |  |  |
|  | 2013 | 84907 | 67547890 | 125.7 (124.85-126.54) | 104.98 (104.26-105.69) |  |  |
|  | 2014 | 83779 | 67745108 | 123.67 (122.83-124.51) | 101.95 (101.25-102.65) |  |  |
|  | 2015 | 82666 | 67907403 | 121.73 (120.9-122.56) | 99.21 (98.52-99.9) |  |  |
|  | 2016 | 81028 | 67941429 | 119.26 (118.44-120.08) | 95.99 (95.32-96.66) |  |  |
|  | 2017 | 81692 | 68179351 | 119.82 (119-120.64) | 95.05 (94.39-95.71) |  |  |
|  | 2018 | 81680 | 68308744 | 119.57 (118.75-120.39) | 93.53 (92.88-94.18) |  |  |
|  | 2019 | 80465 | 68329004 | 117.76 (116.95-118.57) | 91.02 (90.38-91.66) |  |  |
|  | 2020 | 84111 | 68316744 | 123.12 (122.29-123.95) | 94.31 (93.67-94.96) |  |  |
| **South** | 1999 | 186241 | 99164460 | 187.81 (186.96-188.66) | 195.72 (194.83-196.61) | 124.15 (124.01– 124.28) | -3.5% (-3.9 – -3.1) |
|  | 2000 | 183385 | 100236820 | 182.95 (182.11-183.79) | 190.08 (189.21-190.95) |  |  |
|  | 2001 | 179084 | 101849575 | 175.83 (175.02-176.65) | 182.64 (181.8-183.49) |  |  |
|  | 2002 | 175853 | 103150787 | 170.48 (169.68-171.28) | 176.62 (175.8-177.45) |  |  |
|  | 2003 | 170749 | 104380188 | 163.58 (162.81-164.36) | 168.46 (167.66-169.26) |  |  |
|  | 2004 | 161324 | 105883977 | 152.36 (151.62-153.1) | 156.21 (155.45-156.98) |  |  |
|  | 2005 | 159695 | 107479771 | 148.58 (147.85-149.31) | 150.96 (150.22-151.71) |  |  |
|  | 2006 | 151443 | 109076933 | 138.84 (138.14-139.54) | 139.46 (138.76-140.17) |  |  |
|  | 2007 | 144787 | 110688742 | 130.81 (130.13-131.48) | 130.15 (129.48-130.82) |  |  |
|  | 2008 | 143744 | 112184930 | 128.13 (127.47-128.79) | 126 (125.35-126.66) |  |  |
|  | 2009 | 139087 | 113548615 | 122.49 (121.85-123.13) | 118.87 (118.24-119.49) |  |  |
|  | 2010 | 138023 | 114555744 | 120.49 (119.85-121.12) | 115.72 (115.11-116.34) |  |  |
|  | 2011 | 135344 | 116046736 | 116.63 (116.01-117.25) | 109.68 (109.09-110.27) |  |  |
|  | 2012 | 135506 | 117257221 | 115.56 (114.95-116.18) | 106.76 (106.18-107.33) |  |  |
|  | 2013 | 135947 | 118383453 | 114.84 (114.23-115.45) | 104.03 (103.47-104.59) |  |  |
|  | 2014 | 135794 | 119771934 | 113.38 (112.77-113.98) | 101.14 (100.6-101.68) |  |  |
|  | 2015 | 137157 | 121182847 | 113.18 (112.58-113.78) | 99.32 (98.79-99.86) |  |  |
|  | 2016 | 136323 | 122319574 | 111.45 (110.86-112.04) | 96.05 (95.53-96.57) |  |  |
|  | 2017 | 137784 | 123658624 | 111.42 (110.83-112.01) | 94.75 (94.24-95.26) |  |  |
|  | 2018 | 138354 | 124753948 | 110.9 (110.32-111.49) | 92.59 (92.1-93.08) |  |  |
|  | 2019 | 137252 | 125580448 | 109.29 (108.72-109.87) | 89.74 (89.26-90.22) |  |  |
|  | 2020 | 146177 | 126662754 | 115.41 (114.81-116) | 93.46 (92.97-93.95) |  |  |
| **West** | 1999 | 95495 | 62431872 | 152.96 (151.99-153.93) | 178.54 (177.41-179.68) | 109.36 (109.19– 109.52) | -3.9% (-4.2 – -3.6) |
|  | 2000 | 91280 | 63197932 | 144.44 (143.5-145.37) | 167.35 (166.26-168.43) |  |  |
|  | 2001 | 90664 | 64427327 | 140.72 (139.81-141.64) | 161.75 (160.69-162.8) |  |  |
|  | 2002 | 90093 | 65312198 | 137.94 (137.04-138.84) | 157.1 (156.07-158.13) |  |  |
|  | 2003 | 88850 | 66116338 | 134.38 (133.5-135.27) | 151.03 (150.04-152.03) |  |  |
|  | 2004 | 83669 | 66965483 | 124.94 (124.1-125.79) | 139.21 (138.27-140.16) |  |  |
|  | 2005 | 82996 | 67833726 | 122.35 (121.52-123.18) | 133.89 (132.98-134.81) |  |  |
|  | 2006 | 80941 | 68751765 | 117.73 (116.92-118.54) | 127.02 (126.14-127.9) |  |  |
|  | 2007 | 76584 | 69595414 | 110.04 (109.26-110.82) | 116.67 (115.84-117.5) |  |  |
|  | 2008 | 76140 | 70509175 | 107.99 (107.22-108.75) | 112.79 (111.98-113.59) |  |  |
|  | 2009 | 73286 | 71341376 | 102.73 (101.98-103.47) | 105.37 (104.6-106.13) |  |  |
|  | 2010 | 71756 | 71945553 | 99.74 (99.01-100.47) | 101.07 (100.32-101.81) |  |  |
|  | 2011 | 72770 | 72864748 | 99.87 (99.14-100.6) | 98.6 (97.87-99.32) |  |  |
|  | 2012 | 71534 | 73579431 | 97.22 (96.51-97.93) | 93.91 (93.21-94.61) |  |  |
|  | 2013 | 71791 | 74254423 | 96.68 (95.98-97.39) | 91.65 (90.98-92.33) |  |  |
|  | 2014 | 69991 | 75187681 | 93.09 (92.4-93.78) | 86.65 (86-87.3) |  |  |
|  | 2015 | 71820 | 76044679 | 94.44 (93.75-95.14) | 86.27 (85.63-86.91) |  |  |
|  | 2016 | 72423 | 76657000 | 94.48 (93.79-95.16) | 84.7 (84.08-85.33) |  |  |
|  | 2017 | 72812 | 77410622 | 94.06 (93.38-94.74) | 83.42 (82.81-84.04) |  |  |
|  | 2018 | 71943 | 77993663 | 92.24 (91.57-92.92) | 80.16 (79.57-80.75) |  |  |
|  | 2019 | 71475 | 78347268 | 91.23 (90.56-91.9) | 77.92 (77.34-78.49) |  |  |
|  | 2020 | 76166 | 78654756 | 96.84 (96.15-97.52) | 81.18 (80.6-81.77) |  |  |
| Abbreviations: CMR, Crude Mortality Rate; CI, confidence interval; AAMR, age adjusted mortality rate; AAPC, average annual percentage change. | | | | | | | |

**Supplemental Table 3**. **Inflection Points**. Year-to-year inflection points in APC from 1999 to 2020.

| Cumulative | **1999 – 2002** APC -3.8* (95% CI, -5.1 – -2.5), **2002 – 2010** APC -5.3* (95% CI, -5.7 – -4.9), **2010 – 2018** APC -2.7* (95% CI, -3.2 – 2.3), **2018 – 2020** APC 0.7 (95% CI, -2.6 – 4.1) |
| --- | --- |
| Male | **1999 – 2011** APC -4.7* (95% CI, -5.0 – -4.4)  **2011 – 2020** APC -1.8* (95% CI, -2.3 – -1.4) |
| Female | **1999 – 2002** APC -3.7* (95% CI, -5.2 – -2.3), **2002 – 2010** APC -5.9* (95% CI, -6.3 – -5.4), **2010 – 2018** APC -3.5* (95% CI, -4.0 – -3.0), **2018 – 2020** APC 0.1 (95% CI, -3.8 – 4.2) |
| Hispanic | **1999 – 2005** APC -4.3* (95% CI, -5.2 – -3.5), **2005 – 2011** APC -6.4* (95% CI, -7.4 – -5.3), **2011 – 2018** APC -3.0* (95% CI, -3.8 – -2.2), **2018 – 2020** APC 4.4 (95% CI, -0.1 – 9.0) |
| Non-Hispanic | **1999 – 2002** APC -3.8* (95% CI, -5.1 – -2.4), **2002 – 2010** APC -5.2* (95% CI, -5.6 – -4.8), **2010 – 2018** APC -2.7* (95% CI, -3.1 – -2.2), **2018 – 2020** APC 0.5 (95% CI, -2.8 – 4.0) |
| Black | **1999 – 2002** APC -2.6* (95% CI, -3.8 – -1.5), **2002 – 2011** APC -5.6* (95% CI, -5.8 – -5.3), **2011 – 2018** APC -2.9* (95% CI, -3.3 – -2.4), **2018 – 2020** APC 3.9* (95% CI, 1.2 – 6.6) |
| White | **1999 – 2011** APC -4.9* (95% CI, -5.1 – -4.6), **2011 – 2018** APC -2.5* (95% CI, -3.2 – -1.8), **2018 – 2020** APC 0.0 (95% CI, -4.2 – 4.4) |
| Asian/Pacific Islander | **1999 – 2015** APC -4.7* (95% CI, -5.0 – -4.4), **2015 – 2020** APC 0.3 (95% CI, -1.2 – 1.8). |
| American Indian/Alaska Native | **1999 – 2004** APC -2.9* (-5.2 – -0.6),  **2004 – 2008** APC -6.2* (-11.2 – -1.1),  **2008 – 2020** APC -3.2* (-3.8 – -2.6) |
| Metropolitan | **1999 – 2002** APC -3.8* (95% CI, -5.1 – -2.5), **2002 – 2010** APC -5.5* (95% CI, -5.9 – -5.1), **2010 – 2018** APC -2.9* (95% CI, -3.3 – -2.5), **2018 – 2020** APC 0.9 (95% CI, -2.3 – 4.2) |
| Non-metropolitan | **1999 – 2010** APC -4.3* (95% CI, -4.5 – -4.0), **2010 – 2020** APC -1.7* (95% CI, -2.1 – -1.3) |
| Northeast | **1999 – 2002** APC -3.5* (95% CI, -5.0 – -2.0), **2002 – 2011** APC -5.2* (95% CI, -5.6 – -4.9), **2011 – 2018** APC -2.8* (95% CI, -3.5 – -2.2), **2018 – 2020** APC 1.2 (95% CI, -2.8 – 5.3) |
| Midwest | **1999 – 2009** APC -4.9* (95% CI, -5.2 – -4.6), **2009 – 2017** APC -2.9* (95% CI, -3.4 – -2.3), **2017 – 2020** APC -0.3 (95% CI, -2.2 – 1.8) |
| South | **1999 – 2002** APC -3.3* (95% CI, -4.8 – -1.8), **2002 – 2009** APC -5.7* (95% CI, -6.2 – -5.2), **2009 – 2018** APC -2.8* (95% CI, -3.2 – -2.4), **2018 – 2020** APC 0.8 (95% CI, -2.9 – 4.6) |
| West | **1999 – 2012** APC -5.0* (95% CI, -5.3 – -4.7), **2012 – 2020** APC -2.0* (95% CI, -2.7 – -1.4) |

* = indicates APC is significantly different from zero at an α = 0.05 level.

Abbreviations: APC = annual percentage change.

**Supplemental Table 4.**  **The county attributes forming social vulnerability index using American Community Survey, 2014-2018.** Continuous variables reported as medians and interquartile ranges.

| **County Characteristics** | **Median** |
| --- | --- |
| Population estimate | 25,735 (10,918-67,276) |
| Household | 9,869 (4,225-26,044) |
| Housing units | 12,456 (5,473-31,447) |
| **Household composition/disability theme** |  |
| Persons aged ≤ 17 years | 5786 (2,417-15,152) |
| Persons aged ≥ 65 years | 4,623 (2,073-11,842) |
| Civilian non-institutionalized population with a disability | 4,235 (1,742-10,390) |
| Single parent household with children under 18 years | 835 (320-2,257) |
| **Socioeconomic theme** |  |
| Persons below poverty | 3,992 (1,592-9,778) |
| Civilians aged ≥ 16 years unemployed | 667 (255-1,908) |
| Persons aged ≥ 25 years with no high school diploma | 2,492 (992-5,752) |
| Percapita income | 26,245 (22,763-30,116) |
| **Minority status/language theme** |  |
| Minority (all persons except non-Hispanic White | 4,297 (1,206-14,934) |
| Persons aged ≥ 5 years who speak English “less than well” | 175 (40-891) |
| **Housing type transportation theme** |  |
| Housing in structures with 10 or more units | 316 (79-1,558) |
| Mobile homes | 1,489 (574-3,120) |
| Household with no vehicle available | 604 (231-1,579) |
| At household level (occupied housing units) more people than room | 206 (75-564) |
| Persons in group quarters | 634 (188-2,250) |

- The following provides information regarding calculation descriptions: https://www.atsdr.cdc.gov/placeandhealth/svi/documentation/pdf/SVI2018Documentation_01192022_1.pdf

**Supplemental Table 5. Age-adjusted Mortality Rates for Ischemic Heart Diseases Across Social Vulnerability Index Quartiles, 2014 – 2018.** Table depicting the AAMR, RR, and respective 95% CIs for all SVI quartiles.

| Population | SVI – Q1 | SVI – Q2 | SVI – Q3 | SVI – Q4 | RR |
| --- | --- | --- | --- | --- | --- |
| Total – no. (95% CI) | 90.39 (89.62 - 91.16) | 86.34 (86.08 - 86.59) | 96.85 (96.60 - 97.10) | 111.30 (110.98 - 111.62) | 1.23 (0.93-1.63) |
| Male – no. (95% CI) | 115.13 (114.58 - 115.68) | 118.82 (118.35 - 119.28) | 131.64 (131.20 - 132.08) | 148.92 (148.34 - 149.49) | 1.29 (1.01-1.65) |
| Female – no. (95% CI) | 59.49 (59.15 - 59.82) | 60.63 (60.35 - 60.92) | 69.32 (69.05 - 69.60) | 81.63 (81.26 - 81.99) | 1.37 (0.98-1.92) |
| Hispanic – no. (95% CI) | 50.44 (48.98 - 51.89) | 53.94 (52.98 - 54.90) | 64.97 (64.24 - 65.71) | 85.25 (84.59 - 85.91) | 1.69 (1.19-2.39) |
| Non-Hispanic – no. (95% CI) | 84.71 (84.40 - 85.02) | 87.80 (87.53 - 88.06) | 99.54 (99.28 - 99.80) | 117.71 (117.34 - 118.08) | 1.39 (1.05-1.84) |
| Black – no. (95% CI) | 93.54 (91.82 - 95.26) | 92.81 (91.70 - 93.92) | 102.59 (101.84 - 103.34) | 122.60 (121.76 - 123.45) | 1.31 (1.00-1.72) |
| White – no. (95% CI) | 84.82 (84.51 - 85.14) | 88.68 (88.41 - 88.96) | 97.71 (97.44 - 97.98) | 111.82 (111.45 - 112.19) | 1.32 (0.99-1.75) |
| Asian/Pacific Islander – no. (95% CI) | 41.81 (40.47 - 43.15) | 47.52 (46.78 - 48.26) | 57.34 (56.35 - 58.34) | 66.73 (65.60 - 67.86) | 1.60 (1.08-2.35) |
| American Indian/Alaska Native – no. (95% CI) | 60.72 (56.24 - 65.21) | 68.60 (65.46 - 71.74) | 62.02 (59.56 - 64.47) | 78.70 (76.42 - 80.98) | 1.30 (0.93-1.81) |
| Metropolitan – no. (95% CI) | 82.71 (82.38 - 83.05) | 82.84 (82.57 - 83.11) | 93.71 (93.45 - 93.97) | 108.14 (107.78 - 108.51) | 1.31 (0.98-1.74) |
| Non-metropolitan – no. (95% CI) | 90.39 (89.62 - 91.16) | 106.45 (105.72 - 107.18) | 116.73 (116.00 - 117.46) | 122.91 (122.19 - 123.64) | 1.36 (1.04-1.78) |
| Northeast – no. (95% CI) | 89.12 (88.58 - 89.67) | 101.79 (101.13 - 102.45) | 100.31 (99.71 - 100.91) | 121.74 (120.73 - 122.75) | 1.37 (1.04-1.80) |
| Midwest – no. (95% CI) | 87.06 (86.56 - 87.57) | 96.19 (95.64 - 96.75) | 100.69 (100.14 - 101.25) | 128.17 (126.92 - 129.42) | 1.47 (1.12-1.93) |
| South – no. (95% CI) | 75.35 (67.72 - 69.52) | 84.92 (84.47 - 85.38) | 97.77 (97.40 - 98.15) | 112.32 (111.86 - 112.79) | 1.49 (1.11-2.00) |
| West – no. (95% CI) | 68.82 (67.72 - 69.52) | 69.41 (68.98 - 69.85) | 85.83 (85.28 - 86.39) | 102.24 (101.70 - 102.78) | 1.49 (1.09-2.02) |

- All listed values are AAMR per 100,000 population with respective 95% confidence intervals.
- Rate ratios (SVI – Q4 / SVI – Q1) are reported with their respective 95% confidence intervals.
- Abbreviations: AAMR, age-adjusted mortality rate; CI, confidence interval; SVI, social vulnerability index; RR, risk ratio
